# TATES: Efficient Multivariate Genotype-Phenotype Analysis for Genome-Wide Association Studies

**DOI:** 10.1371/journal.pgen.1003235

**Published:** 2013-01-24

**Authors:** Sophie van der Sluis, Danielle Posthuma, Conor V. Dolan

**Affiliations:** 1 Department of Functional Genomics and Department of Clinical Genetics, VU Medical Center, Amsterdam, The Netherlands; 2 Department of Medical Genomics, VU Medical Center, Amsterdam, The Netherlands; 3 Department of Child and Adolescent Psychiatry, Erasmus MC–University Medical Centre, Rotterdam, The Netherlands; 4 Department of Psychology, University of Amsterdam, Amsterdam, The Netherlands; 5 Department of Biological Psychology, VU University Amsterdam, Amsterdam, The Netherlands; University of California San Diego and The Scripps Research Institute, United States of America

## Abstract

To date, the genome-wide association study (GWAS) is the primary tool to identify genetic variants that cause phenotypic variation. As GWAS analyses are generally univariate in nature, multivariate phenotypic information is usually reduced to a single composite score. This practice often results in loss of statistical power to detect causal variants. Multivariate genotype–phenotype methods do exist but attain maximal power only in special circumstances. Here, we present a new multivariate method that we refer to as TATES (Trait-based Association Test that uses Extended Simes procedure), inspired by the GATES procedure proposed by Li et al (2011). For each component of a multivariate trait, TATES combines p-values obtained in standard univariate GWAS to acquire one trait-based p-value, while correcting for correlations between components. Extensive simulations, probing a wide variety of genotype–phenotype models, show that TATES's false positive rate is correct, and that TATES's statistical power to detect causal variants explaining 0.5% of the variance can be 2.5–9 times higher than the power of univariate tests based on composite scores and 1.5–2 times higher than the power of the standard MANOVA. Unlike other multivariate methods, TATES detects both genetic variants that are common to multiple phenotypes and genetic variants that are specific to a single phenotype, i.e. TATES provides a more complete view of the genetic architecture of complex traits. As the actual causal genotype–phenotype model is usually unknown and probably phenotypically and genetically complex, TATES, available as an open source program, constitutes a powerful new multivariate strategy that allows researchers to identify novel causal variants, while the complexity of traits is no longer a limiting factor.

## Author Summary

The genome-wide association study (GWAS) is the primary tool to identify genetic variants that cause phenotypic variation. As GWAS methods are generally univariate in nature, multivariate phenotypic information is usually reduced to a single composite score, which frequently results in a considerable loss of statistical power to detect causal variants. Multivariate genotype–phenotype methods do exist but attain maximal power only in special circumstances. We present a new multivariate method called TATES (Trait-based Association Test that uses Extended Simes procedure). Extensive simulations show that TATES's false positive rate is correct, and that TATES's statistical power to detect causal variants explaining 0.5% of the variance can be 2.5–9 times higher than the power of univariate tests of composite scores and 1.5–2 times higher than the power of the standard MANOVA. Unlike other multivariate methods, TATES uncovers both genetic variants that are common to multiple phenotypes as well as phenotype specific variants. TATES thus provides a more complete view of the genetic architecture of complex traits and constitutes a powerful new multivariate strategy that allows researchers to identify novel causal variants.

## Introduction

Genome-wide association studies (GWAS) are currently the primary tool to identify genetic variants (GVs) underlying phenotypic variation. GWAS are generally univariate in nature, i.e., focus on a single phenotype. This means that researchers, prior to analyses, often reduce available, originally multivariate, phenotypic information (e.g., information on multiple questions from a diagnostic interview or questionnaire, or multiple items in a test) to a single phenotypic composite score, such as a continuous sum score or binary case-control status (the latter is often based on the number of endorsed symptoms, i.e., effectively a dichotomized sum score). Such univariate conceptualisations are consistent with the practical and diagnostic definitions employed in psychology and medicine of traits like depression, cognition, Type I diabetes, and asthma. However, whether they represent informative entities with respect to biological aetiology is questionable [1]. Many acknowledge the possible genetic heterogeneity of psychological and medical traits [2]–[3]. This heterogeneity implies that distinct GVs may give rise to the same univariate trait score, and that the same GV may have different behavioral manifestations, depending on genetic background and environmental exposure. It also implies that phenotypes (e.g., symptoms, items, subtests) may be affected by different GVs. To appreciate this, consider diagnostic indicators of asthma, like spirometric measures, serum total IgE, and fractional exhaled nitric oxide. These measures are phenotypically correlated and all associated with asthma diagnosis, yet their genetic architecture may differ. When GWAS is subsequently conducted on asthma case-control status, however, both the plausible phenotypic and genetic heterogeneity of the trait is discarded. Likewise, depression symptoms like worrying, insomnia, and feeling lonely or irritable, and metabolic syndrome related measures like waist-to-hip ratio, fasting glucose levels, triglycerides, and high-density lipoprotein, are phenotypically correlated yet need not be subject to the same GVs. That is, while the conceptual multidimensionality of traits is often acknowledged in the phenotypic instruments – e.g. by including measures of multiple symptoms for disease traits, or multiple subtests to cover distinguishable dimensions of complex traits (e.g., spatial and verbal ability, memory, and general knowledge in cognition) - this phenotypic resolution is lost when the multivariate phenotypic information is subsequently reduced to a univariate composite score.

**Figure 1:**
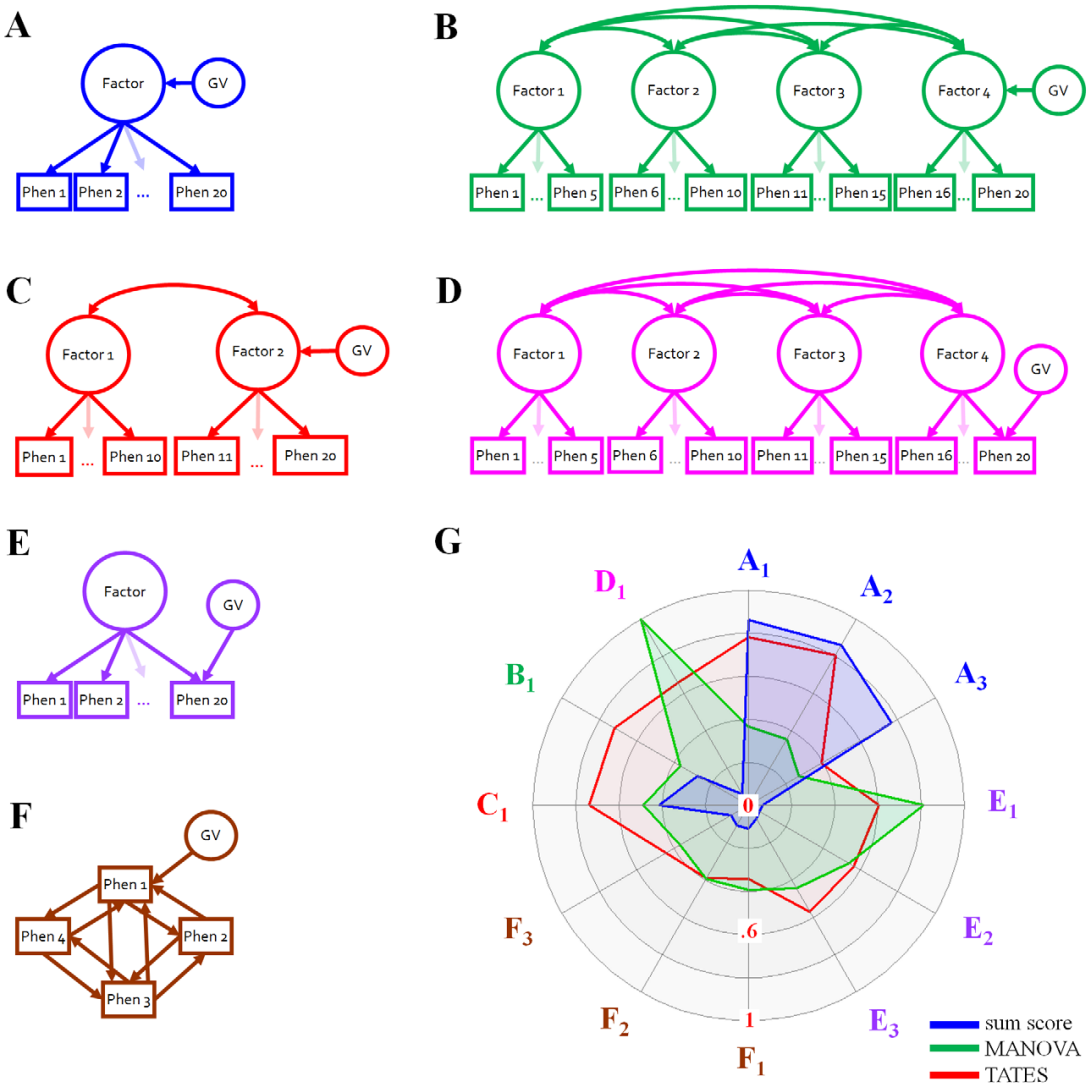
Schematic representation of the simulation settings and results. Schematic representation of the simulation settings (a–f) and radar plot (g) of the power to detect 1 genetic variant (GV) explaining .5% of the phenotypic variance in 12 simulation settings. The power radar plot (power running from 0 (midpoint) to 1 (outer edge)) displays the power for the univariate sum score analyses (blue), MANOVA (green), and TATES (red). The phenotypic correlation structure was either due to one common factor (a,e), multiple underlying latent factors (b,c,d), or a network model (f). Within these phenotypic settings, the GV either affected multiple phenotypes via a common factor (a,b,c), or affect a single phenotype directly (d,e,f). Power results for 12 simulation settings and a GV explaining .5% of the variance are highlighted (g, colour labels corresponds to colour simulation settings; see Tables S1, S2, S3, S4, S5, S6, S7, S8, S9, S10, S11, S12 for more GV effect sizes). Specifically, gA1–3: 1-factor models with GV effect on the factor. gA1: mix of dichotomous, ordinal and continuous phenotypes correlating .36 to .81; gA2: continuous phenotypes correlating .56; gA3: continuous phenotypes correlating .12. gE1–3: 1-factor models with GV effect specific to 1 phenotype. gE1: phenotypes correlate .56 (like gA2); gE2: phenotypes correlate .30; gE3, phenotypes correlate .12 (like gA3). gF1–F3: network models with GV effect specific to 1 phenotype. gF1: phenotypes correlate .56 (like gA2 and gE1); gF2: phenotypes correlate .12 (like gA3 and gE3); gF3: 4 clusters of phenotypes that within clusters correlate .55, and between clusters correlate .13. gC1: 2-factor model, 10 phenotypes per factor, correlating .36–.81 within factors, and a factorial correlation of .5. GV affects only the 2^nd^ factor. gB1: 4-factor model, 5 phenotypes per factor, correlating .81 within factors, and factorial correlations of .1. GV affects only the 4^th^ factor. gD1: like gB1 but GV effect specific to 1 phenotype.

As we often do not know how a causal GV impinges on a phenotype, determining the most informative operationalisation of a trait for gene-finding purposes poses a challenge. Multiple studies [4]–[7] have shown that phenotypic data reduction, such as case-control status phenotypes or sum scores calculated across all distinguishable phenotypes, results in a considerable loss of statistical power to detect GVs in all but the special circumstance that 1) a single phenotypic dimension underlies the variance-covariance structure of the multivariate phenotypes (i.e., single common factor model), and 2) the GV directly affects this dimension (schematic representation Figure 1a). In this ideal unidimensional model, the underlying phenotypic dimension mediates the relationship between the GV and the multivariate phenotypes, and the univariate sum score is a good approximation of this dimension. However, many other genotype-phenotype models are plausible. For instance, the model could be multi-dimensional rather than unidimensional (Figure 1b–1c), and the GV effect could be specific to one of the phenotypes, rather than on the latent dimension (Figure 1d–1e). Recently, the field of psychology has witnessed a shift towards network models, in which relations between individual phenotypes are not believed to result from shared causal latent factors, but rather originate in direct causal influences between phenotypes over time [8]–[10]. For instance, from a network perspective, symptoms like worrying, sleeplessness and agitation are not viewed as manifestations of the latent dimension depression, but as directly and causally related: worrying interferes with sleep, and lack of sleep causes agitation. In such network models, which obviate the need to invoke latent dimensions, each phenotype could be affected by different GVs (Figure 1f). In all these alternative genotype-phenotype models, univariate conceptualisations like sum scores and case-control status result in substantial loss of power to detect underlying GVs.

One way to avoid the potential loss of power associated with univariate conceptualisations of complex heterogeneous traits, is to adopt a multivariate method, which accommodates the originally multivariate nature of the phenotypic measure. Exploratory multivariate strategies, developed in GWAS context, include MultiPhen [11], and canonical correlation analysis [12], which is included in the GWAS software PLINK [13] (as canonical correlation analysis is identical to MANOVA with one GV treated as additive codominant (i.e., covariate), we use the term MANOVA here). MultiPhen uses ordinal regression to regress 0/1/2-coded GVs on a collection of phenotypes of any measurement nature (i.e., continuous, dichotomous, ordinal), and applies one omnibus test to test whether all regression weights in the model are together significantly different from zero. MultiPhen has been shown to outperform MANOVA when minor allele frequency (MAF) is low and the phenotypes are case-control status or non-normally distributed continuous variables [11]. Under most circumstances, however, MultiPhen and MANOVA yield very similar results in terms of power to detect causal GVs.

A drawback of these multivariate methods is that their power depends on the specific configuration of phenotypic correlations and on the location of the GV effect (e.g., on the latent dimension, or specific to one of many correlated phenotypes). For instance, when the ideal model (Figure 1a) holds, MANOVA is decidedly less powerful than univariate analyses based on sum scores. MANOVA, however, easily outperforms the sum score approach when the GV affects only one of multiple strongly correlated variables (e.g., Figure 1d–1f) [4]–[5], [14].

As prior knowledge about the exact location of the GV effect in a multivariate system is usually lacking, a computationally efficient multivariate procedure that performs well in many different circumstances is required to increase the success of future GWAS. Here, we introduce a new multivariate technique called TATES: Trait-based Association Test that uses Extended Simes procedure. TATES is based on the GATES procedure [15], which was developed to combine p-values of individual SNPs located within the same gene into one gene-based p-value P_G_ (where the gene is considered a more attractive unit of analysis for association studies than the SNP because genes are the functional units in the genome). Similarly, for individual phenotypes characterizing a trait (e.g., items or symptoms), TATES combines the p-values obtained in standard univariate GWAS to arrive at a global trait-based p-value P_T_, while correcting for the observed correlational structure between the phenotypes. Here we show that TATES has correct false positive (type-I error) rate, and that TATES picks up both phenotype-specific genetic effects as well as genetic effects that are common to multiple correlated phenotypes. Through extensive simulations, probing a wide variety of genotype-phenotype models, we demonstrate under which circumstances TATES outperforms analyses based on sum scores and MANOVA/MultiPhen with respect to the statistical power to detect causal GVs.

## Results

The TATES method is described in detail in the Materials and Methods section. Briefly, TATES requires the *m*×*n* p-values of the regression of *m* phenotypic variables on *n* GVs, and the *m*×*m* correlation matrix of the phenotypes. The regression of the phenotypes on the GVs can be conducted in standard software packages like PLINK, Mach2dat/qtl, SNPtest, and Gen/ProbABEL [13], [16]–[20], which are fast, facilitate quality control, and can correct for population stratification. For samples that include related individuals, analyses could be conducted using PLINK (where the –mperm option should not vary over the *m* phenotypes to assure that the p-values used in TATES have similar accuracy), *ABLE, PBAT or Merlin-offline [13], [16]–[17], [21]–[22]. For each GV, TATES sorts the *m* p-values ascendingly. To derive from these *m* p-values one trait-based p-value P_T_ for each of the *n* GVs, TATES takes into account that the *m* phenotypes, and thus the *m* p-values, are correlated. In an iterative procedure, TATES weighs the *j* ^th^ p-value in the 1 to *m* sorted p-values with *m*_e_/*m*_ej_, where *m*_e_ is the effective number of independent p-values among all *m* variables, and *m*_ej_ the effective number of p-values among the top *j* p-values. The weight *m*_e_ is a function of *m*, and the sum of those eigenvalues larger than 1 of the *m*×*m* correlation matrix of the p-values. Similarly, *m*_ej_ is a function of *j* and the sum of the eigenvalues larger than 1 based on the *j*×*j* correlation matrix of the top *j* p-values . The correlation matrix of the *m* p-values is approximated from the observed correlation matrix between the *m* phenotypes using a 6^th^ order polynomial (coefficient of determination R^2^ = .992, see Materials and Methods and Figure S1). For each of the *n* GVs, the trait-based TATES p-value P_T_ equals the smallest weighted p-value, with the null-hypothesis that none of the phenotypes is associated with the GV, and the alternative hypothesis that at least one of the phenotypes is associated with the GV. The TATES procedure is implemented in a Fortran 77 program and an R script, both of which are freely available from the website (http://ctglab.nl/software). The Fortran program takes less than 1 minute to calculate the TATES trait-based p-values P_T_ for 12 phenotypes and 437,598 GVs on an ordinary desktop computer with Intel(R) Core(TM)2 Duo CPU 2.99 GHz, RAM 2.94 GB, and 32-bit Windows XP Professional Version 2002.

To study the false positive rate and the power to detect GVs using TATES, we simulated genotype-phenotype data for 2000 subjects and 20 phenotypes (standard normally distributed unless stated otherwise) according to various scenarios that are illustrated in Figure 1a–1f. Specifically, the phenotypic correlation structure was due to one underlying common factor (or dimension, Figure 1a, 1e), multiple underlying common factors (Figure 1b–1d), or to a network model, in which correlations between phenotypes are due to direct, mutual relations between the components (Figure 1f). Within these phenotypic correlational settings, the GV affects multiple phenotypes via the common factor (Figure 1a,b,c), or affects a single component directly (Figure 1d–1f). For each scenario, we simulated GVs (MAF of .50) with effect sizes ranging from 0 to 1% explained variance. The false positive rate was also studied given MAF = .05 and N = 12000. Simulations are described in detail in the Materials and Methods section.

In each scenario, the 20 simulated phenotypes were either a) summed and the sum score was regressed on the GV, b) subjected to a 1-factor model to calculate Thompson's factor scores [23], which were regressed on the GV, c) subjected to a MANOVA with the GV as covariate (canonical correlation analysis), d) subjected to MultiPhen (regressing the GV on all 20 phenotypes in a multivariate ordinal regression model), or e) individually regressed on the GV (using logistic or ordinal regression where appropriate). The last procedure yielded 20 p-values per simulated GV, which were then combined into 1 overall trait-based p-value P_T_ using TATES. In addition, we compared the performance of TATES to that of various other published procedures for combining p-values, limiting our comparison to procedures that, like TATES, do not require permutation, i.e., Fisher's combination test, Lancaster's weighted Fisher test, the Z-transform test, and the original Simes procedure (see Text S1). All data simulations and subsequent analyses were repeated 2000 times. We counted the number of times that the GV effect was detected given α = .05.

The results of all simulated scenarios are presented in detail in Tables S1, S2, S3, S4, S5, S6, S7, S8, S9, S10, S11, S12. The false positive rates of TATES, the sum score and factor scores procedures, MANOVA, and MultiPhen were correct given our simulation settings with both MAF = 50% and MAF = 5%, while the original Simes procedure proved slightly conservative, if the phenotypes were highly correlated. (Note that the false positive rate of MANOVA is known to be inaccurate if the GV has low MAF (.5 or 5%) and the phenotypic data are dichotomous or non-normally distributed [11]). In contrast, the false positive rate of the Fisher combination test, Lancaster's weighted Fisher test, and the Z-transform test, which do not account for correlations between the 20 phenotypes, was often highly inflated (up to 20%, depending on the magnitude of the phenotypic correlations). Power results for these methods are therefore not discussed here (but see Tables S1, S2, S3, S4, S5, S6, S7, S8, S9, S10, S11, S12). Since power results of the factor scores, MultiPhen, and the original Simes procedure were quite similar to those of the sum scores, MANOVA, and TATES, respectively, these are not discussed here (but see Tables S1, S2, S3, S4, S5, S6, S7, S8, S9, S10, S11, S12).

Figure 1g illustrates the power results of 12 selected simulation scenarios for the sum score procedure, MANOVA and TATES, given a GV explaining .5% of the phenotypic variance. As expected [4]–[6], the sum score procedure has excellent power to detect the GV, if the phenotypic data are generated according to a 1-common factor model, and the GV effect is on this factor (Figure 1g: A1–A3). However, if either the location of the GV effect or the data-generating process is different, the power of the univariate sum score procedure drops to levels often <.10 (Figure 1g: B1,C1,D1,E1–3,F1–3). In 9 out of the 12 scenarios we considered, the power of TATES was 2.5 to 9 times higher than the power of the sum score procedure. As expected [4]–[5], [14],the power of MANOVA is especially high if the GV effect is specific to only one of many highly correlated phenotypes (Figure 1g: D1,E1). The power of MANOVA drops if the phenotypic correlations are lower, or if multiple phenotypes are subject to the GV effect. In contrast, TATES is only slightly less powerful than the sum score procedure if the phenotypes correlate substantially (Figure 1g: A1,A2), and clearly more powerful than MANOVA in this condition. TATES outperforms both other procedures if the GV affects multiple, but not all correlated phenotypes (power TATES is 1.5–2 times higher, Figure 1g: B1,C1), and is approximately as good as, or better than, MANOVA, if the GV effect is specific to one of multiple phenotypes that correlate .30 or lower (Figure 1g: E2,E3,F1–3). In 7 of our 12 scenarios, the power of TATES was 1.5 to 2 times higher than the power of MANOVA.

As the original Simes procedure does not take into account the correlations among the p values (originating in the phenotypic correlations), TATES is expected to increasingly outperform Simes as the phenotypic correlations increase. Given low to modest phenotypic correlations, the gain in power acquired with TATES varies from low (1%) to modest (9%) (the latter observed in a 4-factor model with a phenotype-specific GV effect; Table S12). Additional simulations (Tables S13, S14, S15, S16, S17, S18), however, show that, as phenotypic intercorrelations increase in magnitude (.75, .85, .95), the power of TATES can be as much as 10%–19% higher than the power of the Simes procedure, with TATES especially being more powerful when the GV effect is specific to one of multiple correlated phenotype. As TATES is comparable to Simes in computational effort, phenotypes within a trait are almost invariably correlated, and the location of the GV effect is generally unknown (i.e., could be phenotype-specific), one is well-advised to adopt TATES.

Finally, we studied the effect of 10% missingness completely at random (MCAR) or 10% blockwise missingness on the power to detect GVs in three different genotype-phenotype models (see Materials and Methods for details and Tables S19, S20, S21, S22, S23, S24, S25, S26). Power was hardly affected in 1-factor models with the GV effect on the factor. However, if the GV effect was specific to one of the phenotypes (either in factor models or network models), the power of MANOVA usually showed a 5–6% larger drop in power compared to Simes and TATES. Only when the GV effect was specific to a phenotype showing blockwise missingness was the drop in power of Simes and TATES similar to, or even slightly higher (2–3%) than, the power drop observed for MANOVA.

## Discussion

We have presented TATES, a new, computationally feasible multivariate method to test genotype-phenotype relations. The false positive rate of TATES is correct for varying MAF, even if the multiple phenotypes are substantially correlated. Through simulations, we showed that TATES outperforms standard univariate analyses, unless the data-generating process is a unidimensional factor model and the GV affects the factor. TATES is only outperformed by MANOVA in the particular condition that the GV affects only one of multiple strongly correlated phenotypes.

Multivariate genotype-phenotype analyses are important for several reasons. First, most complex traits, such as cognitive ability, personality, problem behavior in humans [24]–[26], and anxiety in mice [27], are multi-dimensional, i.e., multiple common factors are required to describe the variance-covariance structure. Given this multidimensionality, multivariate genotype-phenotype analyses are indicated, as standard univariate analyses cannot accommodate genetic heterogeneity of subdimensions. Second, phenotypically distinguishable subdimensions need not correspond simply to genetic dimensions, and the information to parse a trait into *genetically informative* subdimensions is usually lacking. Consequently, researchers often focus on those GVs that are common to all subdimensions by studying a single, “general” composite measure. A simple, but deficient alternative is to conduct a series of independent univariate association studies without correcting for the dependency between the results caused by the correlations between the phenotypes. TATES offers a simply method to correct for this relatedness, while identifying GVs that are common to multiple phenotypes and GVs that are phenotype specific. As such TATES provides a more complete view of the genetic architecture of complex traits. Third, it is often unclear which phenotype(s) or combination of phenotypes will maximize the probability of unraveling the genetic architecture of a complex trait. For example, in studying a complex trait like schizophrenia, quantitative cognitive traits that are commonly affected in schizophrenia patients (e.g., attention, mental flexibility, memory, sensorimotor processing) may be better candidates for unraveling the genetic architecture of schizophrenia than schizophrenia affection status [28]. Multivariate techniques obviate the need to focus on one phenotype, and help to chart both genetic overlap and genetic uniqueness of related traits. Such information on genetic similarity and dissimilarity of phenotypes may ultimately help uncover the actual disease mechanism.

TATES allows researchers to test their genetic associations efficiently using standard GWAS software. In addition, TATES' reliance on p-value information assures that phenotypes of different measurement levels (e.g., dichotomous, ordinal, continuous) can easily be analyzed simultaneously, and that contrasting effects (i.e., GVs affect some phenotypes positively, some negatively) do not influence the power of the method. Note that the plausibility of contrasting genetic effects does not only depend on the magnitude of the phenotypic correlations and the effect size of the GV (i.e., the correlation matrix between the phenotypes and the GV should remain positive definite), but also on the underlying genotype-phenotype model. For instance, if the true genotype-phenotype model is a 1-factor model with the GV effect on the factor, the direction of the effect of the GV *must* be identical for all phenotypes (assuming that all phenotypes are coded such that higher scores imply higher trait levels). Yet, if the true genotype-phenotype model is a network model, contrasting GV effects are unproblematic from a statistical point of view. Whether contrasting effects are plausible from a biological perspective depends on the actual functional role of the GV. For instance, symptoms like blunted affect and agitated mood can both be positive indicators of depression on a population level, but their biochemical basis may be antagonistic, making contrasting GV effects for these symptoms both statistically and biologically possible.

TATES cannot be used directly to test specific hypotheses concerning the underlying genotype-phenotype model. However, as TATES outputs the p-values from the original univariate GWAS analyses along with TATES' trait-based p-values, further inspection of the pattern of significant univariate tests that drive the significant TATES p-value can be informative. For instance, if a significant TATES p-value is driven by an association with only one of the multiple phenotypes, then the true genotype-phenotype model is unlikely to be a 1-factor model with the GV effect on the factor. The more these phenotype-specific GV effects are observed, the stronger the indication that the trait under study is genetically heterogeneous. This, again, implies that multivariate approaches, in which the heterogeneity is accommodated, are more likely to reveal the genetic architecture of that trait than the standard approach based on univariate composite scores. Furthermore, if one aspires to actually test specific hypotheses concerning the underlying genotype-phenotype model, TATES can be used as a filter to reduce the number of SNPs to a computationally manageable set. The exact location and role of the selected SNPs may then be studied in detail in appropriate multivariate models [4]–[5].

Finally, TATES facilitates the study of the genetic overlap between phenotypes in different domains, for example medical and psychiatric disorders that show high comorbidity, and yet are generally studied separately. Studying behavioural profiles [29] rather than single phenotypes, and phenotypes spanning multiple levels of organisation (e.g., behaviour, morphology, physiology), advances analysis of the “phenome” (the phenotype as a whole, on an organism-wide scale) [30]. Here, TATES is a useful tool, as it is hypothesis- and model-free, and deals with the high phenotypic dimensionality by combining the univariate analyses while correcting for the relatedness between phenomic dimensions. Furthermore, in a highly dimensional phenotypic context, the fact that one does not need to know the underlying data-generating model, or the mechanism causing comorbidity/association between the individual phenotypes in the analysis, is an attractive feature of TATES.

To summarize, TATES is an efficient multivariate method for combining p-values across different, correlated phenotypes in genotype-phenotype analyses. In the context of gene-finding studies, TATES allows researchers to test genetic associations without a priori data reduction or commitment to one phenotypic or genetic model. As the actual phenotypic and genetic architecture of traits is usually unknown and probably complex, an exploratory multivariate procedure like TATES provides a viable and, as simulations show, powerful new strategy.

## Materials and Methods

### TATES

Suppose *m* phenotypes are measured as indicators of one trait, e.g., individual symptoms within a disorder, items within a test, or multiple measures of one trait using different instruments (e.g., open-field test, a light-dark box, and an elevated plus maze to measure anxiety in mice). Rather than combining these *m* phenotypes into one general phenotype, we test the association between all *m* phenotypes and all *n* genotyped genetic variants (GVs) using a statistically appropriate method (e.g., linear or logistic regression). Let p(1)…p(m) be the ascending p-values of the *m* phenotypes for a given GV. TATES combines within each GV the *m* phenotype-specific p-values to obtain one overall trait-based p-value P_T_ as follows:
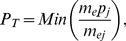
(1)where *m*_e_ denotes the effective number of independent p-values of all *m* phenotypes for a given GV, and *m*_ej_ the effective number of p-values among the top *j* p-values, where *j* runs from 1 to *m*, and *p*_j_ denotes the *j* ^th^ p-value in the list of ordered p-values. P_T_ is thus the smallest weighted p-value, associated with the null hypothesis that none of the phenotypes is associated with the GV, and the alternative hypothesis that at least one of the phenotypes is associated with the GV.

Following Li et al [15], we obtain an estimate of the effective number of p-values *m*_ej_ through a correction based on eigenvalue decomposition of the *m*×*m* correlation matrix *ρ* between the p-values associated with the *m* phenotypes. The effective number of p-values *m*_ej_ for the top *j* p-values is calculated as:
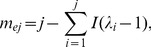
(2)where *j* is the number of top *j* p-values, λ_i_ denotes the *i* ^th^ eigenvalue, and *I*( λ_i_−1) is an indicator function taking on value 0 if λ_i_≤1 and 1 if λ_i_>1. That is, the effective number of p-values *m*_ej_ is calculated as the observed number of p-values *j* minus the sum of the difference between the eigenvalues λ_i_ and 1 for those eigenvalues λ_i_>1. If the *j* phenotypes are all uncorrelated, then all *j* eigenvalues equal 1, and *m*_ej_ = *j*−0 = *j*. In contrast, if the *j* phenotypes are perfectly correlated, then the first eigenvalue equals *j*, and the other eigenvalues equal 0, rendering *m*_ej_ = *j*−(*j*−1) = 1 (i.e., *j* perfectly correlated phenotypes represent only 1 unique unit of information). In practice, phenotypes show intercorrelations of variable magnitude (but not 0 or 1), so the effective number of p-values *m*_ej_ will usually be smaller than *j*, but greater than 1. Note that *m*_e_ is equal to *m*_ej_ for the case that *j* = *m*, i.e., when the selection of top phenotypes covers all phenotypes.

### Approximation p-value correlation matrix

The *m*×*m* correlation matrix *ρ* between the p-values is not observed in practice. Following Li et al [15], we used simulation to show that matrix *ρ* can be accurately approximated through the *m*×*m* correlation matrix *r* between the phenotypes. We simulated 55 continuous standard normally distributed phenotypes whose intercorrelations ranged between −.90 and .90, and a GV (MAF = .5) that was simulated to be unrelated to the 55 phenotypes. The association between the GV and all phenotypes was tested, yielding 55 p-values, and this simulation was run 10,000 times. We then calculated, across the 10,000 simulations, the mean pair-wise correlations between the 55 phenotypes (i.e., (55*55−55)/2 = 1485 correlations), and the mean pair-wise correlations between the p-values. Regressing the vector of correlations between the p-values on the vector of correlations between the phenotypes, we obtain the 6^th^ order polynomial *ρ* = −0.0008−0.0023*r*+0.6226*r* ^2^+0.0149*r* ^3^+0.1095*r* ^4^−0.0219*r* ^5^+0.2179*r* ^6^ (coefficient of determination R^2^ = .992; see Figure S1), allowing accurate approximation of the correlations between the p-values from the observed correlations between the phenotypes. The thus obtained matrix *ρ* is subjected to the eigenvalue decomposition in Eq. 2.

### Simulations

#### General settings

All simulations concerned N = 2000 subjects and 20 standard normally distributed phenotypes (N∼(0,1)), unless stated otherwise. Simulated GVs (MAF = .5) explained 0 to 1% (with steps of .01) of the variance in either the latent factor, or in a specific phenotype (see below). All simulations were repeated N_sim_ = 2000 times. Simulations and analyses were conducted in *R* [31].

#### Factor models

For *m* phenotypes and *k* common factors, data were simulated according to the model:
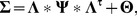
(3)where **Σ** denotes the *m*×*m* variance-covariance matrix between the phenotypes, **Λ** is the *m*×*k* matrix of factor loadings (^t^ denotes matrix transpose), **Ψ** is the *k*×*k* variance-covariance matrix between the common factors, and **Θ** is the *m*×*m* diagonal matrix of residual variances (i.e., the part of the phenotypic variance that is not explained by latent factors). In simulations with multiple factors (k>1), we maintained simple structure, i.e., each phenotype is related to only one factor.

Sum scores calculated across all *m* phenotypes are only sufficient statistics (exhaustively summarizing all information available in the individual phenotypes) if a) all correlations between the phenotypes are explained by 1 latent factor, b) all phenotypes have identical factor loadings, and c) all phenotypes have identical residual variances [6] (a so-called Rasch model [32]). In the case of 1 factor models (Figure 1a and 1e), we thus chose to simulate phenotypic data according to Rasch models, as this represents the most favorable condition for the univariate sum score method. Factor loadings ranged between .75 (corresponding to .75^2^ = .56% explained variance by the factor, and 1−.75^2^ = .4375% residual variance; *A2*, *E1* in Figure 1g), .55 (.30% explained; *E2*) and .35 (.12% explained; *A3*, *E3*). With these settings, intercorrelations between all *m* phenotypes are .56, .30, or .12, respectively. The GV effect was then either modeled on the factor (Figure 1a; Figure 1g *A1–A3*), affecting via the factor all phenotypes defining the factor (in which case the GV effect is weighted by the factor loadings; the lower the factor loading, the smaller the GV effect on a phenotype), or specifically on the residual variance of one phenotype (Figure 1e; Figure 1g *E1–E3*). Note that a sum score only summarizes both phenotypic *and* genetic information exhaustively if the GV affects the factor; if the GV affects one phenotype specifically, the sum score operationalisation is not sufficient from a genetic perspective.

A special case was simulation *A1*, in which we simulated a 1-factor model for a mix of dichotomous, ordinal (3 categories), and continuous phenotypes with factor loadings ranging from .60 to .90, to show that TATES also works well for phenotypes of different measurement levels. In this specific simulation, the correlation matrix between the phenotypes, used to approximate the correlations between the p-values, was mixed with the type of correlation (Pearson, polyserial, polychoric) depending on the measurement levels of the phenotypes involved.

In the 2-factor model (*C1*), each factor was indicated by 10 phenotypes, with factor loadings ranging from .60 to .90 within each factor, a factorial correlation of .5, and the GV affecting the 2^nd^ factor only. In 4-factor models (*B1*, *D1*), each factor was indicated by 5 phenotypes, with factor loadings of .90 within each factor, factorial correlations of .10, and the GV affecting either the 4^th^ factor (*B1*), or one phenotype specifically (*D1*).

#### Network models

All network simulations concerned a stationary network, i.e., assuming that mutual interactions between phenotypes have over time resulted in a stable variance-covariance matrix. Assuming *m* phenotypes, stationary network data were created according to the model:
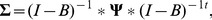
(4)where **Σ** denotes the *m*×*m* variance-covariance matrix between the phenotypes, **I** is a *m*×*m* identity matrix, and **B** is a full *m*×*m* matrix containing the regression parameters β of all the phenotypes on each other (e.g., element **B**[*i,j*] contains the regression parameter β of phenotype i on phenotype j). The diagonal of the matrix **B** was set to 0, implying absence of self-activation of the phenotypes (i.e., the phenotypes do not affect themselves). **Ψ** is a *m*×*m* diagonal matrix containing the variances of all phenotypes conditional on the effects of the other phenotypes. In all network simulations, the GV was only associated to the first phenotype in the network (Figure 1f). Note, however, that the GV effect spreads throughout the network as all phenotypes in the network were directly or indirectly interrelated. To assure convergence of our network models (i.e., simulation settings result in stable systems), we checked the sufficient condition that the largest eigenvalue of B*B ^t^ is smaller than 1 [33].

Two types of networks were simulated. First (*F1*,*F2*), all regression weights in matrix **B** were set to .04202, or .08187, resulting in phenotypic intercorrelations of .56 or .12, respectively, i.e., the phenotypic variance-covariance matrix of the network simulations mimics the phenotypic variance-covariance matrix of two Rasch models discussed above(*A2*/*E1*,*A3/E3*). Second (*F3*), a network was simulated with 4 clusters of strongly associated phenotypes (correlation = .55), and weaker associations between clusters (correlation = .13).

Importantly, data generated according to a network or factor model can have the very same variance-covariance structure, despite different underlying, data-generating processes. Consequently, even if a 1-factor model describes the phenotypic data well, this does not guarantee that the 1-factor model is the actual data-generating model. This realization is relevant for univariate GWAS where factor analytic results are often taken as indication that reduction of the multivariate data to sum scores is justified. In reality, however, such reduction is only justified if the *data-generating process* is a unidimensional factor model, but not if the data-generating process is a network model.

### TATES versus original Simes

To determine the circumstances in which TATES outperforms the original Simes procedure, we conducted six additional simulations. While the original Simes procedure corrects for the *observed* number of p-values, TATES corrects for the *effective* number of p-values, by taking the correlations between the p-values into account. The difference in terms of power between Simes and TATES is thus expected to be larger as the correlations between the p-values (phenotypes) are stronger (i.e., the effective number becomes smaller).

To illustrate the difference in power between TATES and Simes, we simulated phenotypic data according to 1-factor Rasch models, with factor loadings of .8660, .9220, or .9747, indicating correlations of .75, .85 and .95 between the phenotypes, respectively. The GV effect was modeled either on the latent factor (like Figure 1a; Tables S13, S14, S15), or directly on one of the 20 phenotypes (like Figure 1e; Tables S16, S17, S18).

### Missingness and power

To study the effect of missingness in the phenotypic data on the power to detect GVs, we conducted eight simulation studies in which we studied two types of missingness in five different genotype-phenotype models. The effect of missingness completely at random (MCAR) was studied by simulating data in which each of the 20 simulated phenotypes had 10% missingness distributed randomly across individuals. With 2000 subjects and 20 phenotypes, this results in ∼4000 missing values (i.e., 10% of the total of 40000 observations). In addition, we studied the effect of blockwise missingness; 400 randomly selected subjects in each simulated file had valid data only for the first 10 of 20 phenotypes (e.g., comparable to the situation that data of two samples are combined: in sample 1 (N = 1600), a full 20-item questionnaire is administered, while in sample 2 (N = 400), the abbreviated version of 10 items is administered). This results again in 4000 missing values, i.e., the amount of missingness is the same across the two missingness scenarios, but the distribution is different.

The effect of these two types of missingness was studied in three genotype-phenotype models: 1) 1-factor Rasch model with the GV effect on the factor (Figure 1a; Tables S19, S20), 2) 1-factor Rasch model with the GV effect specific to one phenotype (Figure 1e; specific phenotype not showing blockwise missingness; Tables S21, S22, or showing blockwise missingness; Table S23), 3) network model with the GV effect specific to one phenotype (Figure 1f; specific phenotype not showing blockwise missingness; Tables S24, S25, or showing blockwise missingness; Table S26). In all these models, the 20 phenotypes correlated .56 (power results including missingness can thus be compared to power results concerning the same models without missingness presented in Tables S2, S4 and S7). Note that equal correlations between all phenotypes represents the ideal situation in which all phenotypes are equally reliable, i.e., the effect of the missingness only depends on the pattern of missingness, not e.g. on the reliability of the individual phenotypes.

In subsequent analyses, missingness was handled in two ways. The missing values were either imputed using mean imputation (i.e., missing values are imputed with the sample mean of the appropriate phenotype). This type of imputation, which was done for MANOVA, sum score, Simes and TATES, is standard in MultiPhen [11] and canonical correlation analysis in Plink [13]. Alternatively, the analyses were based on all available valid data. The sum score was then calculated as a weighted sum (i.e., the sum of all available data, divided by the total number of available data). For Simes and TATES, the univariate tests were based on all available data, and the p-values, now due to the missingness based on different sample sizes, were combined as usual. (Whether a correction is required to deal with the fact that the p-values are based on different sample sizes, is open to debate. In theory, the test statistic, and thus the p-value, already take N into account. In practice, however, a procedure that weights for the sample size can be more powerful [34]. We tried one type of weighting for Simes and TATES, in which each p-value was weighted by df_max_/df_j_, where df_max_ denotes the maximal number of degrees of freedom (i.e., sample size) of the 20 simulated phenotypes, and df_j_ denotes the number of degrees of freedom for the *j* ^th^ phenotype in the set of 1…20. This way, the p-value belonging to the largest sample was weighted by df_max_/df_max_ = 1, while the other p-values were weighted by df_max_/df_j_ and as df_j_ is always <df_max_ the weight is thus >1, i.e., p-values derived from small samples were adjusted upwards and are therefore less likely to be the minimal p-value chosen by Simes or TATES.)

MANOVA was not conducted on all available data because in standard MANOVA, cases are excluded listwise, resulting in a very low sample size when missingness is MCAR. In theory, fitting MANOVA on the raw data using Full Information Maximum Likelihood (FIML) is possible in software like LISREL, Mx, or Mplus [33], [35]–[36], but this is time consuming in a genome-wide context. Here, we chose to stick to the common practice of MultiPhen [11] and Plink [13], which is mean imputation.

## Supporting Information

Figure S1 The relationship between correlations between phenotypes *r* (x-axis) and correlations between p-values (y-axis) obtained in the regression of 55 phenotypes on a genetic variant. Simulations (Nsim = 10,000, Nsubjects = 2000, Nitem = 55, Nsnp = 1, see Materials and Methods for details) show that this relationship can be accurately described by a 6^th^ order polynomial (coefficient of determination R^2^ = .992).
(DOC)

Table S1 Power to detect genetic variant (GV) in 1-factor model with phenotypes of different measurement levels and GV effect on the factor (Figure 1g. A1).
(DOC)

Table S2 Power to detect GV in 1-factor Rasch model with factor loadings of .75 (phenotypic intercorrelations .56), and GV effect on the factor (Figure 1g. A2).
(DOC)

Table S3 Power to detect GV in 1-factor Rasch model with factor loadings of .35 (phenotypic intercorrelations .30), and GV effect on the factor (Figure 1g. A3).
(DOC)

Table S4 Power to detect GV in 1-factor Rasch model with factor loadings of .75 (phenotypic intercorrelations .56), and GV effect specific to phenotype (Figure 1g. E1).
(DOC)

Table S5 Power to detect GV in 1-factor Rasch model with factor loadings of .55 (phenotypic intercorrelations .30), and GV effect specific to phenotype (Figure 1g. E1).
(DOC)

Table S6 Power to detect GV in 1-factor Rasch model with factor loadings of .35 (phenotypic intercorrelations .12), and GV effect specific to phenotype (Figure 1g. E1).
(DOC)

Table S7 Power to detect GV in a network model with all phenotypic intercorrelations .56, and GV effect specific to phenotype (Figure 1g. F1).
(DOC)

Table S8 Power to detect GV in a network model with all phenotypic intercorrelations .12, and GV effect specific to phenotype (Figure 1g. F2).
(DOC)

Table S9 Power to detect GV in a network model with cluster of phenotypes correlating .55 within, and .13 between clusters, and GV effect specific to phenotype (Figure 1g. F3).
(DOC)

Table S10 Power to detect GV (MAF = .5) in a 2-factor model, with 10 phenotypes per factor, factor loadings within factors ranging between .6 and .9, factorial correlations of .5, and GV effect specific to the 2^nd^ factor (Figure 1g. C1).
(DOC)

Table S11 Power to detect GV (MAF = .5) in a 4-factor model, with 5 phenotypes per factor, factor loadings of .9, factorial correlations of .1, and GV effect specific to the 4^th^ factor (Figure 1g. B1).
(DOC)

Table S12 Power to detect GV in a 4-factor model, with 5 phenotypes per factor, factor loadings of .9, factorial correlations of .1, and GV effect specific to one phenotype (Figure 1g. D1).
(DOC)

Table S13 Power to detect GV in 1-factor Rasch model with factor loadings of .866 (phenotypic intercorrelations .75), and GV effect on the factor.
(DOC)

Table S14 Power to detect GV in 1-factor Rasch model with factor loadings of .922 (phenotypic intercorrelations .85), and GV effect on the factor.
(DOC)

Table S15 Power to detect GV in 1-factor Rasch model with factor loadings of .9747 (phenotypic intercorrelations .95), and GV effect on the factor.
(DOC)

Table S16 Power to detect GV in 1-factor Rasch model with factor loadings of .866 (phenotypic intercorrelations .75), and GV effect specific to one phenotype.
(DOC)

Table S17 Power to detect GV in 1-factor Rasch model with factor loadings of .922 (phenotypic intercorrelations .85), and GV effect specific to one phenotype.
(DOC)

Table S18 Power to detect GV in 1-factor Rasch model with factor loadings of .9747 (phenotypic intercorrelations .95), and GV effect specific to one phenotype.
(DOC)

Table S19 Power to detect GV in 1-factor Rasch model with factor loadings of .75 (phenotypic intercorrelations .56), 10% missingness completely at random, and GV effect on the factor.
(DOC)

Table S20 Power to detect GV in 1-factor Rasch model with factor loadings of .75 (phenotypic intercorrelations .56), 10% blockwise missingness, and GV effect on the factor.
(DOC)

Table S21 Power to detect GV in 1-factor Rasch model with factor loadings of .75 (phenotypic intercorrelations .56), 10% missingness completely at random, and GV effect specific to one phenotype.
(DOC)

Table S22 Power to detect GV in 1-factor Rasch model with factor loadings of .75 (phenotypic intercorrelations .56), 10% blockwise missingness, and GV effect specific to one phenotype *NOT* showing blockwise missingness.
(DOC)

Table S23 Power to detect GV in 1-factor Rasch model with factor loadings of .75 (phenotypic intercorrelations .56), 10% blockwise missingness, and GV effect specific to one phenotype showing blockwise missingness.
(DOC)

Table S24 Power to detect GV in a network model with all phenotypic intercorrelations .56, 10% missingness completely at random, and GV effect specific to one phenotype.
(DOC)

Table S25 Power to detect GV in a network model with all phenotypic intercorrelations .56, 10% blockwise missingness, and GV effect specific to one phenotype *NOT* showing blockwise missingness.
(DOC)

Table S26 Power to detect GV in a network model with all phenotypic intercorrelations .56, 10% blockwise missingness, and GV effect specific to one phenotype showing blockwise missingness.
(DOC)

Text S1 Additional information on alternative methods to combine p-value information (Fisher combination test, Lancaster's weighted Fisher test, Z-transform test, original Simes test) and on calculation of confidence intervals for the p-values from the simulations.
(DOC)

## References

[1] Corvin A, Craddock N, Sullivan PF (2010) Genome-wide association studies: a primer. Psychol Med 40: 1063–1077. 19895722 10.1017/S0033291709991723 PMC4181332

[2] McClellan J, King MC (2010) Genetic heterogeneity in human disease. Cell 141: 210–217. 20403315 10.1016/j.cell.2010.03.032

[3] Dowell RD, Ryan O, Jansen A, Cheung D, Agarwala S, et al (2010) Genotype to phenotype: a complex problem. Science 328: 469–469. 20413493 10.1126/science.1189015 PMC4412269

[4] Medland S, Neale MC (2010) An integrated phenomic approach to multivariate allelic association. Eur J Hum Genet 18: 233–239. 19707246 10.1038/ejhg.2009.133 PMC2807471

[5] Minica CC, Boomsma DI, van der Sluis S, Dolan CV (2010) Genetic Association in Multivariate Phenotypic Data: Power in Five Models. Twin Res Hum Genet 13: 525–543. 21142929 10.1375/twin.13.6.525

[6] Van der Sluis S, Verhage M, Posthuma D, Dolan CV (2010) Phenotypic Complexity, Measurement Bias, and Poor Phenotypic Resolution Contribute to the Missing Heritability Problem in Genetic Association Studies. Plos One 5: e13929. 21085666 10.1371/journal.pone.0013929 PMC2978099

[7] Van der Sluis S, Posthuma D, Nivard MG, Verhage M, Dolan CV (2012) Power in GWAS: lifting the curse of the clinical cut-off. Mol Psych doi:10.1038/mp.2012.65. 10.1038/mp.2012.65 22614290

[8] Van der Maas HLJ, Dolan CV, Grasman RPPP, Wicherts JM, Huizenga HM, et al (2006) A Dynamic model of general intelligence: the positive manifold of intelligence by mutualism. Psychol Rev 113: 842–861. 17014305 10.1037/0033-295X.113.4.842

[9] Cramer AOJ, Waldorp LJ, van der Maas HLJ, Borsboom D (2010) Comorbidity: a network perspective. Behav Brain Sci 33: 137–193. 20584369 10.1017/S0140525X09991567

[10] Borsboom D, Cramer AOJ, Schmittmann VD, Epskamp S, Waldorp LJ (2011) The small world of psychopathology. Plos One 6: e27407. 22114671 10.1371/journal.pone.0027407 PMC3219664

[11] O'Reilly PF, Hoggart CJ, Pomyen Y, Calboli FCF, Elliott P, et al (2012) MultiPhen: Joint model of multiple phenotypes can increase discovery in GWAS. Plos One 7: e34861. 22567092 10.1371/journal.pone.0034861 PMC3342314

[12] Ferreira MAR, Purcell SM (2009) A multivariate test of association. Bioinformatics 25: 132–133. 19019849 10.1093/bioinformatics/btn563

[13] Purcell S, Neale B, Todd-Brown K, Thomas L, Ferreira MAR, et al (2007) PLINK: a tool set for whole-genome association and population-based linkage analyses. Am J Hum Genet 81: 559–575. 17701901 10.1086/519795 PMC1950838

[14] Cole DA, Maxwell SE, Avrey RD, Salas E (1994) How the power of MANOVA can both increase and decrease as a function of the intercorrelations among the dependent variables. Psychol Bull 115: 465–474.

[15] Li M-X, Gui H-S, Kwan JSH, Sham PC (2011) GATES: a rapid and powerful gene-based association test using extended Simes procedure. Am J Hum Genet 88: 283–293. 21397060 10.1016/j.ajhg.2011.01.019 PMC3059433

[16] Aulchenko YS, Ripke S, Isaacs A, van Duijn CM (2007) GenABEL: an R library for genome-wide association analysis. Bioinformatics 23: 1294–1296. 17384015 10.1093/bioinformatics/btm108

[17] Aulchenko YS, Struchalin MV, van Duijn CM (2010) ProbABEL package for genome-wide association analysis of imputed data. BMC Bioinformatics 11: 134. 20233392 10.1186/1471-2105-11-134 PMC2846909

[18] Li Y, Willer CJ, Ding J, Scheet P, Abecasis GR (2010) MaCH: using sequence and genotype data to estimate haplotypes and unobserved genotypes. Genet Epidemiol 34: 816–834. 21058334 10.1002/gepi.20533 PMC3175618

[19] Li Y, Willer CJ, Sanna S, Abecasis GR (2009) Genotype Imputation. Annu Rev Genomics Hum Genet 10: 387–406. 19715440 10.1146/annurev.genom.9.081307.164242 PMC2925172

[20] Marchini J, Howie B, Myers S, McVean G, Donnelly P (2007) A new multipoint method for genome-wide association studies via imputation of genotypes. Nat Genet 39: 906–913. 17572673 10.1038/ng2088

[21] Abecasis GR, Cherny SS, Cookson WO, Cardon LR (2002) Merlin-rapid analysis of dense genetic maps using sparse gene flow trees. Nat Genet 30: 97–101. 11731797 10.1038/ng786

[22] Lange C, DeMeo D, Silverman EK, Weiss ST, Laird NM (2004) PBAT: Tools for family-based association studies. Am J Hum Genet 74: 367–369. 14740322 10.1086/381563 PMC1181934

[23] Lawley DN, Maxwell AE (1971) Factor analysis as a statistical method. London: Butterworth.

[24] Carroll JB (1993) Human Cognitive abilities: A survey of factor analytic studies. Cambridge University press.

[25] Achenbach TM (1991) Manual for the Child Behavior Checklist/4–18. Burlington, VT: University of Vermont, Department of Psychiatry.

[26] Digman JM (1997) Higher-order factors of the big five. J Pers Soc Psychol 73: 1246–1256. 9418278 10.1037//0022-3514.73.6.1246

[27] Henderson ND, Turri MG, DeFries JC, Flint J (2004) QTL analysis of multiple behavioural measures of anxiety in mice. Behav Genet 34: 267–293. 14990867 10.1023/B:BEGE.0000017872.25069.44

[28] Brzustowics LM, Bassett AS (2008) Phenotype matters: The case for careful characterization of relevant traits. Am J Psychiat 165: 1096–1098. 18765489 10.1176/appi.ajp.2008.08060897 PMC3276589

[29] Bloss CS, Schiabor KM, Schork NJ (2010) Human behavioral informatics in genetic studies of neuropsychiatric disease: multi-variate profile-based analysis. Brain Res Bull 83: 177–188. 20433907 10.1016/j.brainresbull.2010.04.012 PMC2941546

[30] Houle D, Govindaraju DR, Omholt S (2010) Phenomics: the next challenge. Nat Rev Genet 11: 855–866. 21085204 10.1038/nrg2897

[31] R Development Core Team (2011) R: A language and environment for statistical computing. R Foundation for Statistical Computing, Vienna, Austria. ISBN 3-900051-07-0, URL http://www.R-project.org/.

[32] Rasch G (1980) Probabilistic models for some intelligence and attainment tests. Chicago: The University of Chicago Press.

[33] Jöreskog KG, Sörbom D (1996–2001) LISREL 8 User's Reference Guide, SSI Scientific Software International. Suite. USA

[34] Whitlock MC (2005) Combining probability from independent tests: the weighted Z-method is superior to Fisher's approach. J Evolution Biol 18: 1368–1373. 10.1111/j.1420-9101.2005.00917.x 16135132

[35] Neale MC, Boker SM, Xie G, Maes HH (2006) Mx: statistical modeling, 7th edn. Department of Psychiatry, VCU, Richmond.

[36] Muthén LK, Muthén BO (1998–2012) Mplus User's Guide. Seventh Edition. Los Angeles, CA: Muthén & Muthén

