# Supplementary material for: TATES: Efficient Multivariate Genotype-Phenotype Analysis for Genome-Wide Association Studies
Source: PLoS Genet. 2013 Jan 24;9(1):e1003235. doi: 10.1371/journal.pgen.1003235 (PMC3554627; doi:10.1371/journal.pgen.1003235)
Supplement: Figure S1 — The relationship between correlations between phenotypes r (x-axis) and correlations between p-values (y-axis) obtained in the regression of 55 phenotypes on a genetic variant. Simulations (Nsim = 10,000, Nsubjects = 2000, Nitem = 55, Nsnp = 1, see Materials and Methods for details) show that this relationship can be accurately described by a 6th order polynomial (coefficient of determination R2 = .992). (DOC) [file pgen.1003235.s001.doc]

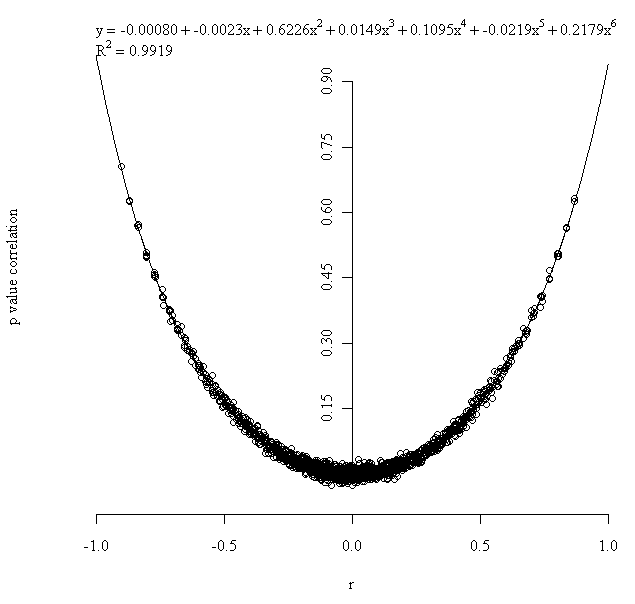


**Supplementary Fig. 1**: The relationship between correlations between phenotypes *r* (x-axis) and correlations between p-values (y-axis) obtained in the regression of 55 phenotypes on a genetic variant. Simulations (Nsim=10,000, Nsubjects=2000, Nitem=55, Nsnp=1, see **Online Methods** for details) show that this relationship can be accurately described by a 6^th^ order polynomial (coefficient of determination R^2^=.992)
