# Supplementary material for: TATES: Efficient Multivariate Genotype-Phenotype Analysis for Genome-Wide Association Studies
Source: PLoS Genet. 2013 Jan 24;9(1):e1003235. doi: 10.1371/journal.pgen.1003235 (PMC3554627; doi:10.1371/journal.pgen.1003235)
Supplement: Table S1 — Power to detect genetic variant (GV) in 1-factor model with phenotypes of different measurement levels and GV effect on the factor (Figure 1g. A1). (DOC) [file pgen.1003235.s002.doc]

| Table S1.  Power to detect GV (MAF=.5) in 1-factor model with phenotypes of different measurement levels and GV effect on the factor (Fig. 1g. A1) | | | | | | | | | |
| --- | --- | --- | --- | --- | --- | --- | --- | --- | --- |
|  | sum | factor | MANOVA | Fisher | Fisher-L | Z | Simes | TATES | MultiPhen |
| 0% | 0.0545 | 0.0545 | 0.0510 | 0.1305 | 0.1840 | 0.1845 | 0.0505 | 0.0580 | 0.0535 |
| 0.1% | 0.2785 | 0.2820 | 0.0980 | 0.4340 | 0.5105 | 0.5110 | 0.2010 | 0.2240 | 0.0975 |
| 0.2% | 0.4890 | 0.4980 | 0.1450 | 0.6515 | 0.7170 | 0.7165 | 0.3705 | 0.3985 | 0.1685 |
| 0.3% | 0.6525 | 0.6550 | 0.2090 | 0.7905 | 0.8485 | 0.8485 | 0.5355 | 0.5600 | 0.2350 |
| 0.4% | 0.7815 | 0.7890 | 0.3060 | 0.8730 | 0.9115 | 0.9115 | 0.6770 | 0.6955 | 0.2950 |
| 0.5% | 0.8605 | 0.8680 | 0.3660 | 0.9355 | 0.9580 | 0.9580 | 0.7585 | 0.7810 | 0.3755 |
| 0.6% | 0.9105 | 0.9120 | 0.4610 | 0.9540 | 0.9705 | 0.9705 | 0.8460 | 0.8595 | 0.4905 |
| 0.7% | 0.9570 | 0.9600 | 0.5415 | 0.9805 | 0.9870 | 0.9870 | 0.9135 | 0.9220 | 0.5525 |
| 0.8% | 0.9750 | 0.9770 | 0.6050 | 0.9900 | 0.9940 | 0.9940 | 0.9440 | 0.9535 | 0.6395 |
| 0.9% | 0.9855 | 0.9855 | 0.6825 | 0.9940 | 0.9960 | 0.9960 | 0.9640 | 0.9675 | 0.7055 |
| 1% | 0.9920 | 0.9930 | 0.7530 | 0.9965 | 0.9985 | 0.9985 | 0.9785 | 0.9795 | 0.7630 |
|  |  |  |  |  |  |  |  |  |  |
| False positive rate for MAF=.05 (N=12000) | | | | | | | | | |
| 0% | 0.0525 | 0.0525 | 0.054 | 0.1195 | 0.1845 | 0.1865 | 0.0435 | 0.054 | .0445 |
|  |  |  |  |  |  |  |  |  |  |
| Note: Power to detect a GV that explains varying amounts of variance in 1 latent factor.  Abbreviations are: *sum*: analysis of the sum across all phenotypes; *factor*: analysis of the factors score across all phenotypes calculated as Thompson scores; *MANOVA*: multivariate-analysis of variance with all phenpotypes as dependent variables; *Fisher*: Fisher combination test; *Fisher-L*: Lancaster’s weighted Fisher test; *Z*: Z-transform test; *Simes*: original Simes test; *TATES*: trait-based association test using extended Simes procedure.  Nphenotype=20, Nsubject=2000, Nsimulation=2000. | | | | | | | | | |
