# Supplementary material for: TATES: Efficient Multivariate Genotype-Phenotype Analysis for Genome-Wide Association Studies
Source: PLoS Genet. 2013 Jan 24;9(1):e1003235. doi: 10.1371/journal.pgen.1003235 (PMC3554627; doi:10.1371/journal.pgen.1003235)
Supplement: Table S2 — Power to detect GV in 1-factor Rasch model with factor loadings of .75 (phenotypic intercorrelations .56), and GV effect on the factor (Figure 1g. A2). (DOC) [file pgen.1003235.s003.doc]

| Table S2  Power to detect GV (MAF=.5) in 1-factor Rasch model with factor loadings of .75 (phenotypic intercorrelations .56), and GV effect on the factor (Fig. 1g. A2) | | | | | | | | | |
| --- | --- | --- | --- | --- | --- | --- | --- | --- | --- |
|  | sum | factor | MANOVA | Fisher | Fisher-L | Z | Simes | TATES | MultiPhen |
| 0% | 0.0510 | 0.0500 | 0.0520 | 0.1390 | 0.2075 | 0.2075 | 0.0385 | 0.0445 | 0.0525 |
| 0.1% | 0.2785 | 0.2775 | 0.0905 | 0.4715 | 0.5505 | 0.5490 | 0.2075 | 0.2270 | 0.0955 |
| 0.2% | 0.5075 | 0.5075 | 0.1560 | 0.6835 | 0.7630 | 0.7625 | 0.4040 | 0.4210 | 0.1685 |
| 0.3% | 0.6780 | 0.6765 | 0.2155 | 0.8215 | 0.8610 | 0.8605 | 0.5725 | 0.5910 | 0.2335 |
| 0.4% | 0.7990 | 0.7975 | 0.3115 | 0.9020 | 0.9315 | 0.9315 | 0.7005 | 0.7185 | 0.3160 |
| 0.5% | 0.8595 | 0.8590 | 0.3570 | 0.9465 | 0.9675 | 0.9675 | 0.7920 | 0.8070 | 0.3860 |
| 0.6% | 0.9135 | 0.9140 | 0.4420 | 0.9675 | 0.9785 | 0.9790 | 0.8545 | 0.8675 | 0.4855 |
| 0.7% | 0.9605 | 0.9600 | 0.5410 | 0.9870 | 0.9930 | 0.9930 | 0.9200 | 0.9290 | 0.5395 |
| 0.8% | 0.9695 | 0.9695 | 0.6205 | 0.9950 | 0.9960 | 0.9960 | 0.9435 | 0.9495 | 0.6560 |
| 0.9% | 0.9895 | 0.9890 | 0.6895 | 0.9990 | 0.9985 | 0.9985 | 0.9695 | 0.9710 | 0.7120 |
| 1% | 0.9945 | 0.9945 | 0.7565 | 0.9990 | 0.9995 | 0.9995 | 0.9825 | 0.9845 | 0.7795 |
|  |  |  |  |  |  |  |  |  |  |
| False positive rate for MAF=.05 (N=12000) | | | | | | | | | |
| 0% | 0.0475 | 0.0475 | 0.0605 | 0.147 | 0.2025 | 0.2025 | 0.039 | 0.0425 | .0495 |
|  |  |  |  |  |  |  |  |  |  |
| Note: Power to detect a GV that explains varying amounts of variance in 1 latent factor.  Abbreviations are: *sum*: analysis of the sum across all phenotypes; *factor*: analysis of the factors score across all phenotypes calculated as Thompson scores; *MANOVA*: multivariate-analysis of variance with all phenpotypes as dependent variables; *Fisher*: Fisher combination test; *Fisher-L*: Lancaster’s weighted Fisher test; *Z*: Z-transform test; *Simes*: original Simes test; *TATES*: trait-based association test using extended Simes procedure.  Nphenotype =20, Nsubject=2000, Nsimulation=2000. | | | | | | | | | |
