# Supplementary material for: TATES: Efficient Multivariate Genotype-Phenotype Analysis for Genome-Wide Association Studies
Source: PLoS Genet. 2013 Jan 24;9(1):e1003235. doi: 10.1371/journal.pgen.1003235 (PMC3554627; doi:10.1371/journal.pgen.1003235)
Supplement: Table S3 — Power to detect GV in 1-factor Rasch model with factor loadings of .35 (phenotypic intercorrelations .30), and GV effect on the factor (Figure 1g. A3). (DOC) [file pgen.1003235.s004.doc]

| Table S3  Power to detect GV (MAF=.5) in 1-factor Rasch model with factor loadings of .35 (phenotypic intercorrelations .12), and GV effect on the factor (Fig. 1g. A3) | | | | | | | | | |
| --- | --- | --- | --- | --- | --- | --- | --- | --- | --- |
|  | sum | factor | MANOVA | Fisher | Fisher-L | Z | Simes | TATES | MultiPhen |
| 0% | 0.0565 | 0.0535 | 0.0540 | 0.0410 | 0.0675 | 0.0680 | 0.0570 | 0.0580 | 0.0580 |
| 0.1% | 0.2065 | 0.2065 | 0.0790 | 0.1365 | 0.1855 | 0.1860 | 0.1065 | 0.1080 | 0.0955 |
| 0.2% | 0.3985 | 0.3975 | 0.1295 | 0.2705 | 0.3675 | 0.3690 | 0.1805 | 0.1815 | 0.1235 |
| 0.3% | 0.5555 | 0.5560 | 0.1630 | 0.4185 | 0.4965 | 0.4995 | 0.2510 | 0.2520 | 0.1950 |
| 0.4% | 0.6745 | 0.6760 | 0.2235 | 0.5365 | 0.6130 | 0.6160 | 0.3200 | 0.3205 | 0.2155 |
| 0.5% | 0.7660 | 0.7655 | 0.2705 | 0.6305 | 0.7055 | 0.7070 | 0.3910 | 0.3920 | 0.2960 |
| 0.6% | 0.8255 | 0.8230 | 0.3520 | 0.7140 | 0.7785 | 0.7810 | 0.4765 | 0.4780 | 0.3685 |
| 0.7% | 0.8940 | 0.8950 | 0.4080 | 0.8025 | 0.8430 | 0.8435 | 0.5570 | 0.5570 | 0.4295 |
| 0.8% | 0.9295 | 0.9290 | 0.4690 | 0.8545 | 0.8920 | 0.8925 | 0.6125 | 0.6145 | 0.4930 |
| 0.9% | 0.9480 | 0.9480 | 0.5430 | 0.8795 | 0.9120 | 0.9140 | 0.6685 | 0.6700 | 0.5530 |
| 1% | 0.9640 | 0.9630 | 0.5870 | 0.9215 | 0.9415 | 0.9425 | 0.7120 | 0.7130 | 0.6090 |
|  |  |  |  |  |  |  |  |  |  |
| Note: Power to detect a GV that explains varying amounts of variance in 1 latent factor.  Abbreviations are: *sum*: analysis of the sum across all phenotypes; *factor*: analysis of the factors score across all phenotypes calculated as Thompson scores; *MANOVA*: multivariate-analysis of variance with all phenpotypes as dependent variables; *Fisher*: Fisher combination test; *Fisher-L*: Lancaster’s weighted Fisher test; *Z*: Z-transform test; *Simes*: original Simes test; *TATES*: trait-based association test using extended Simes procedure.  Nphenotype =20, Nsubject=2000, Nsimulation=2000. | | | | | | | | | |
