# Supplementary material for: TATES: Efficient Multivariate Genotype-Phenotype Analysis for Genome-Wide Association Studies
Source: PLoS Genet. 2013 Jan 24;9(1):e1003235. doi: 10.1371/journal.pgen.1003235 (PMC3554627; doi:10.1371/journal.pgen.1003235)
Supplement: Table S4 — Power to detect GV in 1-factor Rasch model with factor loadings of .75 (phenotypic intercorrelations .56), and GV effect specific to phenotype (Figure 1g. E1). (DOC) [file pgen.1003235.s005.doc]

| Table S4  Power to detect GV (MAF=.5) in 1-factor Rasch model with factor loadings of .75 (phenotypic intercorrelations .56), and GV effect specific to phenotype (Fig. 1g. E1) | | | | | | | | | |
| --- | --- | --- | --- | --- | --- | --- | --- | --- | --- |
|  | sum | factor | MANOVA | Fisher | Fisher-L | Z | Simes | TATES | MultiPhen |
| 0% | 0.0590 | 0.0590 | 0.0565 | 0.1470 | 0.2085 | 0.2070 | 0.0385 | 0.0460 | 0.0440 |
| 0.1% | 0.0520 | 0.0530 | 0.1695 | 0.1665 | 0.2265 | 0.2265 | 0.0880 | 0.1025 | 0.1700 |
| 0.2% | 0.0450 | 0.0450 | 0.3140 | 0.1565 | 0.2175 | 0.2170 | 0.1620 | 0.1815 | 0.3640 |
| 0.3% | 0.0530 | 0.0525 | 0.5490 | 0.2025 | 0.2460 | 0.2475 | 0.3220 | 0.3505 | 0.5350 |
| 0.4% | 0.0500 | 0.0495 | 0.6985 | 0.2220 | 0.2610 | 0.2620 | 0.4370 | 0.4620 | 0.7150 |
| 0.5% | 0.0670 | 0.0640 | 0.8140 | 0.2610 | 0.2900 | 0.2915 | 0.5695 | 0.6030 | 0.8375 |
| 0.6% | 0.0535 | 0.0535 | 0.9075 | 0.2600 | 0.2705 | 0.2725 | 0.7000 | 0.7285 | 0.9050 |
| 0.7% | 0.0590 | 0.0595 | 0.9460 | 0.2860 | 0.2790 | 0.2815 | 0.7860 | 0.8105 | 0.9510 |
| 0.8% | 0.0555 | 0.0565 | 0.9785 | 0.2970 | 0.2700 | 0.2730 | 0.8645 | 0.8830 | 0.9765 |
| 0.9% | 0.0640 | 0.0640 | 0.9900 | 0.3595 | 0.2830 | 0.2855 | 0.9010 | 0.9130 | 0.9870 |
| 1% | 0.0625 | 0.0630 | 0.9935 | 0.3695 | 0.2855 | 0.2870 | 0.9390 | 0.9515 | 0.9950 |
|  |  |  |  |  |  |  |  |  |  |
| False positive rate for MAF=.05 (N=12000) | | | | | | | | | |
| 0% | 0.0475 | 0.0475 | 0.0605 | 0.147 | 0.2025 | 0.2025 | 0.039 | 0.0425 | .0495 |
|  |  |  |  |  |  |  |  |  |  |
| Note: Power to detect a GV that explains varying amounts of variance in one phenotype specifically in the context of a 1-factor model.  Abbreviations are: *sum*: analysis of the sum across all phenotypes; *factor*: analysis of the factors score across all phenotypes calculated as Thompson scores; *MANOVA*: multivariate-analysis of variance with all phenpotypes as dependent variables; *Fisher*: Fisher combination test; *Fisher-L*: Lancaster’s weighted Fisher test; *Z*: Z-transform test; *Simes*: original Simes test; *TATES*: trait-based association test using extended Simes procedure.  Nphenotype =20, Nsubject=2000, Nsimulation=2000. | | | | | | | | | |
