# Supplementary material for: TATES: Efficient Multivariate Genotype-Phenotype Analysis for Genome-Wide Association Studies
Source: PLoS Genet. 2013 Jan 24;9(1):e1003235. doi: 10.1371/journal.pgen.1003235 (PMC3554627; doi:10.1371/journal.pgen.1003235)
Supplement: Table S5 — Power to detect GV in 1-factor Rasch model with factor loadings of .55 (phenotypic intercorrelations .30), and GV effect specific to phenotype (Figure 1g. E1). (DOC) [file pgen.1003235.s006.doc]

| Table S5  Power to detect GV (MAF=.5) in 1-factor Rasch model with factor loadings of .55 (phenotypic intercorrelations .30), and GV effect specific to phenotype (Fig. 1g. E2) | | | | | | | | | |
| --- | --- | --- | --- | --- | --- | --- | --- | --- | --- |
|  | sum | factor | MANOVA | Fisher | Fisher-L | Z | Simes | TATES | MultiPhen |
| 0% | 0.0485 | 0.0490 | 0.0565 | 0.0825 | 0.1260 | 0.1265 | 0.0450 | 0.0465 | 0.0500 |
| 0.1% | 0.0565 | 0.0565 | 0.1085 | 0.1040 | 0.1510 | 0.1515 | 0.0955 | 0.0985 | 0.1315 |
| 0.2% | 0.0575 | 0.0575 | 0.2275 | 0.1285 | 0.1625 | 0.1630 | 0.1905 | 0.1950 | 0.2190 |
| 0.3% | 0.0525 | 0.0515 | 0.3280 | 0.1510 | 0.1820 | 0.1855 | 0.3095 | 0.3180 | 0.3280 |
| 0.4% | 0.0540 | 0.0535 | 0.4535 | 0.1825 | 0.1935 | 0.1970 | 0.4450 | 0.4525 | 0.4540 |
| 0.5% | 0.0570 | 0.0595 | 0.5365 | 0.2115 | 0.2025 | 0.2055 | 0.5560 | 0.5650 | 0.5750 |
| 0.6% | 0.0560 | 0.0560 | 0.6615 | 0.2715 | 0.2280 | 0.2335 | 0.7165 | 0.7205 | 0.6830 |
| 0.7% | 0.0560 | 0.0590 | 0.7505 | 0.2860 | 0.2110 | 0.2160 | 0.7955 | 0.7990 | 0.7605 |
| 0.8% | 0.0620 | 0.0610 | 0.7745 | 0.2880 | 0.2165 | 0.2225 | 0.7920 | 0.7955 | 0.8340 |
| 0.9% | 0.0675 | 0.0685 | 0.8770 | 0.3870 | 0.2290 | 0.2365 | 0.9070 | 0.9095 | 0.8865 |
| 1% | 0.0675 | 0.0685 | 0.9255 | 0.457 | 0.2490 | 0.2560 | 0.9380 | 0.9405 | 0.9165 |
|  |  |  |  |  |  |  |  |  |  |
| Note: Power to detect a GV that explains varying amounts of variance in one phenotype specifically in the context of a 1-factor model.  Abbreviations are: *sum*: analysis of the sum across all phenotypes; *factor*: analysis of the factors score across all phenotypes calculated as Thompson scores; *MANOVA*: multivariate-analysis of variance with all phenpotypes as dependent variables; *Fisher*: Fisher combination test; *Fisher-L*: Lancaster’s weighted Fisher test; *Z*: Z-transform test; *Simes*: original Simes test; *TATES*: trait-based association test using extended Simes procedure.  Nphenotype =20, Nsubject=2000, Nsimulation=2000. | | | | | | | | | |
