# Supplementary material for: TATES: Efficient Multivariate Genotype-Phenotype Analysis for Genome-Wide Association Studies
Source: PLoS Genet. 2013 Jan 24;9(1):e1003235. doi: 10.1371/journal.pgen.1003235 (PMC3554627; doi:10.1371/journal.pgen.1003235)
Supplement: Table S6 — Power to detect GV in 1-factor Rasch model with factor loadings of .35 (phenotypic intercorrelations .12), and GV effect specific to phenotype (Figure 1g. E1). (DOC) [file pgen.1003235.s007.doc]

| Table S6  Power to detect GV (MAF=.5) in 1-factor Rasch model with factor loadings of .35 (phenotypic intercorrelations .12), and GV effect specific to phenotype (Fig. 1g. E3) | | | | | | | | | |
| --- | --- | --- | --- | --- | --- | --- | --- | --- | --- |
|  | sum | factor | MANOVA | Fisher | Fisher-L | Z | Simes | TATES | MultiPhen |
| 0% | 0.0590 | 0.0590 | 0.0440 | 0.0305 | 0.0585 | 0.0590 | 0.0540 | 0.0540 | 0.0520 |
| 0.1% | 0.0590 | 0.0590 | 0.1075 | 0.0585 | 0.1015 | 0.1040 | 0.1130 | 0.1145 | 0.1010 |
| 0.2% | 0.0560 | 0.0550 | 0.1715 | 0.0895 | 0.1145 | 0.1150 | 0.2005 | 0.2005 | 0.1780 |
| 0.3% | 0.0620 | 0.0625 | 0.2445 | 0.1225 | 0.1360 | 0.1375 | 0.3150 | 0.3160 | 0.2635 |
| 0.4% | 0.0625 | 0.0605 | 0.3375 | 0.1405 | 0.1390 | 0.1440 | 0.4530 | 0.4535 | 0.3790 |
| 0.5% | 0.070 | 0.0710 | 0.4430 | 0.1920 | 0.1645 | 0.1695 | 0.5680 | 0.5700 | 0.4510 |
| 0.6% | 0.0705 | 0.0720 | 0.5450 | 0.2685 | 0.1915 | 0.1985 | 0.7050 | 0.7060 | 0.5350 |
| 0.7% | 0.0730 | 0.0750 | 0.6475 | 0.3205 | 0.2010 | 0.2050 | 0.7910 | 0.7915 | 0.6335 |
| 0.8% | 0.0695 | 0.0765 | 0.6345 | 0.3240 | 0.2015 | 0.2100 | 0.7875 | 0.7875 | 0.7150 |
| 0.9% | 0.0955 | 0.0940 | 0.7680 | 0.4355 | 0.2095 | 0.2225 | 0.9085 | 0.9085 | 0.7710 |
| 1% | 0.0775 | 0.0760 | 0.8335 | 0.5015 | 0.2490 | 0.2595 | 0.9375 | 0.9390 | 0.8280 |
|  |  |  |  |  |  |  |  |  |  |
| Note: Power to detect a GV that explains varying amounts of variance in one phenotype specifically in the context of a 1-factor model.  Abbreviations are: *sum*: analysis of the sum across all phenotypes; *factor*: analysis of the factors score across all phenotypes calculated as Thompson scores; *MANOVA*: multivariate-analysis of variance with all phenpotypes as dependent variables; *Fisher*: Fisher combination test; *Fisher-L*: Lancaster’s weighted Fisher test; *Z*: Z-transform test; *Simes*: original Simes test; *TATES*: trait-based association test using extended Simes procedure.  Nphenotype =20, Nsubject=2000, Nsimulation=2000. | | | | | | | | | |
