# Supplementary material for: TATES: Efficient Multivariate Genotype-Phenotype Analysis for Genome-Wide Association Studies
Source: PLoS Genet. 2013 Jan 24;9(1):e1003235. doi: 10.1371/journal.pgen.1003235 (PMC3554627; doi:10.1371/journal.pgen.1003235)
Supplement: Table S7 — Power to detect GV in a network model with all phenotypic intercorrelations .56, and GV effect specific to phenotype (Figure 1g. F1). (DOC) [file pgen.1003235.s008.doc]

| Table S7  Power to detect GV (MAF=.5) in a network model with all phenotypic intercorrelations .56, and GV effect specific to phenotype (Fig. 1g. F1) | | | | | | | | | |
| --- | --- | --- | --- | --- | --- | --- | --- | --- | --- |
|  | sum | factor | MANOVA | Fisher | Fisher-L | Z | Simes | TATES | MultiPhen |
| 0% | 0.0495 | 0.0490 | 0.0450 | 0.1435 | 0.2040 | 0.2040 | 0.0380 | 0.0450 | 0.0480 |
| 0.1% | 0.0630 | 0.0630 | 0.0945 | 0.1810 | 0.2345 | 0.2335 | 0.0625 | 0.0690 | 0.0975 |
| 0.2% | 0.0745 | 0.0730 | 0.1420 | 0.1905 | 0.2500 | 0.2505 | 0.1000 | 0.1165 | 0.1545 |
| 0.3% | 0.0800 | 0.0800 | 0.2250 | 0.2115 | 0.2705 | 0.2710 | 0.1575 | 0.1770 | 0.2130 |
| 0.4% | 0.0955 | 0.0945 | 0.3075 | 0.2485 | 0.2970 | 0.2975 | 0.2370 | 0.2625 | 0.3145 |
| 0.5% | 0.1090 | 0.1090 | 0.3900 | 0.2690 | 0.3180 | 0.3180 | 0.3115 | 0.3415 | 0.4105 |
| 0.6% | 0.1110 | 0.1105 | 0.4445 | 0.2905 | 0.3380 | 0.3385 | 0.4115 | 0.4360 | 0.4540 |
| 0.7% | 0.1320 | 0.1305 | 0.5575 | 0.3175 | 0.3510 | 0.3515 | 0.4870 | 0.5105 | 0.5465 |
| 0.8% | 0.1535 | 0.1530 | 0.6100 | 0.3460 | 0.3765 | 0.3775 | 0.5575 | 0.5850 | 0.6290 |
| 0.9% | 0.1840 | 0.1805 | 0.6915 | 0.3850 | 0.3990 | 0.4005 | 0.6350 | 0.6600 | 0.6905 |
| 1% | 0.1500 | 0.1475 | 0.7360 | 0.3725 | 0.3945 | 0.3955 | 0.6875 | 0.7140 | 0.7490 |
|  |  |  |  |  |  |  |  |  |  |
| False positive rate for MAF=.05 (N=12000) | | | | | | | | | |
| 0% | 0.0485 | 0.0485 | 0.056 | 0.138 | 0.203 | 0.2025 | 0.0345 | 0.041 | .0445 |
|  |  |  |  |  |  |  |  |  |  |
| Note: Simulations based on 20 phenotypes adhering to a network model with all beta’s equal to .04202, resulting in intercorrelations of .56 in the stationary correlation matrix. The GV-effect was modeled on only the first phenotype only.  Abbreviations are: *sum*: analysis of the sum across all items/symptoms; *factor*: analysis of the factors score across all items calculated as Thompson scores; *MANOVA*: multivariate-analysis of variance with all items as dependent variables; *Fisher*: Fisher combination test; *Fisher-L*: Lancaster’s weighted Fisher test; *Z*: Z-transform test; *Z_w_*: weighted Z-transform test; *Simes*: original Simes test; *TATES*: trait-based association test using extended Simes procedure.  Nphenotype =20, Nsubject=2000, Nsimulation=2000. | | | | | | | | | |
