# Supplementary material for: TATES: Efficient Multivariate Genotype-Phenotype Analysis for Genome-Wide Association Studies
Source: PLoS Genet. 2013 Jan 24;9(1):e1003235. doi: 10.1371/journal.pgen.1003235 (PMC3554627; doi:10.1371/journal.pgen.1003235)
Supplement: Table S8 — Power to detect GV in a network model with all phenotypic intercorrelations .12, and GV effect specific to phenotype (Figure 1g. F2). (DOC) [file pgen.1003235.s009.doc]

| Table S8  Power to detect GV (MAF=.5) in a network model with all phenotypic intercorrelations .12, and GV effect specific to phenotype (Fig. 1g. F2) | | | | | | | | | |
| --- | --- | --- | --- | --- | --- | --- | --- | --- | --- |
|  | sum | factor | MANOVA | Fisher | Fisher-L | Z | Simes | TATES | MultiPhen |
| 0% | 0.0515 | 0.0495 | 0.0450 | 0.0290 | 0.0650 | 0.0665 | 0.0475 | 0.0480 | 0.0555 |
| 0.1% | 0.0590 | 0.0610 | 0.0960 | 0.0570 | 0.1090 | 0.1075 | 0.0800 | 0.0805 | 0.0975 |
| 0.2% | 0.0755 | 0.0770 | 0.1440 | 0.0815 | 0.1485 | 0.1500 | 0.1220 | 0.1230 | 0.1490 |
| 0.3% | 0.0845 | 0.0900 | 0.2180 | 0.1335 | 0.1830 | 0.1840 | 0.1975 | 0.2000 | 0.2220 |
| 0.4% | 0.0930 | 0.0995 | 0.3115 | 0.1860 | 0.2380 | 0.2400 | 0.3140 | 0.3150 | 0.2935 |
| 0.5% | 0.1065 | 0.1115 | 0.3940 | 0.2305 | 0.2645 | 0.2680 | 0.3895 | 0.3910 | 0.3755 |
| 0.6% | 0.1070 | 0.1150 | 0.4480 | 0.2675 | 0.2985 | 0.3070 | 0.4570 | 0.4605 | 0.4560 |
| 0.7% | 0.1220 | 0.1310 | 0.5460 | 0.3315 | 0.3370 | 0.3400 | 0.5405 | 0.5445 | 0.5665 |
| 0.8% | 0.1570 | 0.1655 | 0.6190 | 0.4110 | 0.3855 | 0.3930 | 0.6120 | 0.6140 | 0.6485 |
| 0.9% | 0.1675 | 0.1815 | 0.6985 | 0.4885 | 0.4425 | 0.4535 | 0.7215 | 0.7220 | 0.6870 |
| 1% | 0.1800 | 0.1915 | 0.7685 | 0.5535 | 0.4730 | 0.4810 | 0.7720 | 0.7730 | 0.7610 |
|  |  |  |  |  |  |  |  |  |  |
| Note: Simulations based on 20 phenotypes adhering to a network model with all beta’s equal to .08187, resulting in intercorrelations of .12 in the stationary correlation matrix. The GV-effect was modeled on only the first phenotype only.  Abbreviations are: *sum*: analysis of the sum across all items/symptoms; *factor*: analysis of the factors score across all items calculated as Thompson scores; *MANOVA*: multivariate-analysis of variance with all items as dependent variables; *Fisher*: Fisher combination test; *Fisher-L*: Lancaster’s weighted Fisher test; *Z*: Z-transform test; *Z_w_*: weighted Z-transform test; *Simes*: original Simes test; *TATES*: trait-based association test using extended Simes procedure.  Nphenotype =20, Nsubject=2000, Nsimulation=2000. | | | | | | | | | |
