# Supplementary material for: TATES: Efficient Multivariate Genotype-Phenotype Analysis for Genome-Wide Association Studies
Source: PLoS Genet. 2013 Jan 24;9(1):e1003235. doi: 10.1371/journal.pgen.1003235 (PMC3554627; doi:10.1371/journal.pgen.1003235)
Supplement: Table S9 — Power to detect GV in a network model with cluster of phenotypes correlating .55 within, and .13 between clusters, and GV effect specific to phenotype (Figure 1g. F3). (DOC) [file pgen.1003235.s010.doc]

| Table S9  Power to detect GV (MAF=.5) in a network model with cluster of phenotypes correlating .55 within, and .13 between clusters, and GV effect specific to phenotype (Fig. 1g. F3) | | | | | | | | | |
| --- | --- | --- | --- | --- | --- | --- | --- | --- | --- |
|  | sum | factor | MANOVA | Fisher | Fisher-L | Z | Simes | TATES | MultiPhen |
| 0% | 0.0595 | 0.0585 | 0.0620 | 0.0910 | 0.1500 | 0.1515 | 0.0455 | 0.0565 | 0.0425 |
| 0.1% | 0.0690 | 0.0675 | 0.0955 | 0.1160 | 0.1635 | 0.1645 | 0.0845 | 0.0990 | 0.0950 |
| 0.2% | 0.0735 | 0.0735 | 0.1380 | 0.1470 | 0.1910 | 0.1920 | 0.1410 | 0.1575 | 0.1610 |
| 0.3% | 0.0865 | 0.0845 | 0.2285 | 0.1920 | 0.2385 | 0.2405 | 0.2210 | 0.2415 | 0.2285 |
| 0.4% | 0.1020 | 0.1035 | 0.2915 | 0.2200 | 0.2490 | 0.2510 | 0.3160 | 0.3400 | 0.3075 |
| 0.5% | 0.0945 | 0.0935 | 0.3675 | 0.2375 | 0.2485 | 0.2555 | 0.4180 | 0.4405 | 0.3970 |
| 0.6% | 0.1175 | 0.1185 | 0.4615 | 0.3065 | 0.3065 | 0.3115 | 0.5095 | 0.5350 | 0.4770 |
| 0.7% | 0.1340 | 0.1365 | 0.5255 | 0.3310 | 0.3125 | 0.3160 | 0.6065 | 0.6290 | 0.5610 |
| 0.8% | 0.1295 | 0.1280 | 0.6205 | 0.3770 | 0.3315 | 0.3360 | 0.6960 | 0.7145 | 0.6415 |
| 0.9% | 0.1525 | 0.1535 | 0.6890 | 0.4140 | 0.3595 | 0.3640 | 0.7705 | 0.7855 | 0.7030 |
| 1% | 0.1660 | 0.1670 | 0.7440 | 0.4475 | 0.3750 | 0.3820 | 0.8275 | 0.8405 | 0.7710 |
|  |  |  |  |  |  |  |  |  |  |
| False positive rate for MAF=.05 (N=12000) | | | | | | | | | |
| 0% | 0.0495 | 0.051 | 0.0495 | 0.083 | 0.1285 | 0.1295 | 0.042 | 0.049 | .0505 |
|  |  |  |  |  |  |  |  |  |  |
| Note: Simulations based on 20 phenotypes adhering to a network model with 4 clusters of 5 phenotypes correlating .55 within, and .13 between clusters. The GV-effect was modeled on only the first phenotype only.  Abbreviations are: *sum*: analysis of the sum across all items/symptoms; *factor*: analysis of the factors score across all items calculated as Thompson scores; *MANOVA*: multivariate-analysis of variance with all items as dependent variables; *Fisher*: Fisher combination test; *Fisher-L*: Lancaster’s weighted Fisher test; *Z*: Z-transform test; *Z_w_*: weighted Z-transform test; *Simes*: original Simes test; *TATES*: trait-based association test using extended Simes procedure.  Nphenotype =20, Nsubject=2000, Nsimulation=2000. | | | | | | | | | |
