# Supplementary material for: TATES: Efficient Multivariate Genotype-Phenotype Analysis for Genome-Wide Association Studies
Source: PLoS Genet. 2013 Jan 24;9(1):e1003235. doi: 10.1371/journal.pgen.1003235 (PMC3554627; doi:10.1371/journal.pgen.1003235)
Supplement: Table S10 — Power to detect GV (MAF = .5) in a 2-factor model, with 10 phenotypes per factor, factor loadings within factors ranging between .6 and .9, factorial correlations of .5, and GV effect specific to the 2nd factor (Figure 1g. C1). (DOC) [file pgen.1003235.s011.doc]

| Table S10  Power to detect GV (MAF=.5) in a 2-factor model, with 10 phenotypes per factor, factor loadings within factors ranging between .6 and .9, factorial correlations of .5, and GV effect specific to the 2^nd^ factor (Fig. 1g. C1) | | | | | | | | | |
| --- | --- | --- | --- | --- | --- | --- | --- | --- | --- |
|  | sum | factor | MANOVA | Fisher | Fisher-L | Z | Simes | TATES | MultiPhen |
| 0% | 0.0515 | 0.0505 | 0.0590 | 0.1185 | 0.1770 | 0.1780 | 0.0380 | 0.0450 | 0.0590 |
| 0.1% | 0.1275 | 0.1375 | 0.1085 | 0.3365 | 0.4120 | 0.4120 | 0.1595 | 0.1805 | 0.1030 |
| 0.2% | 0.2040 | 0.2400 | 0.1905 | 0.5235 | 0.5915 | 0.5940 | 0.3110 | 0.3340 | 0.1980 |
| 0.3% | 0.2915 | 0.3225 | 0.2880 | 0.6915 | 0.7425 | 0.7430 | 0.4715 | 0.4965 | 0.2850 |
| 0.4% | 0.3625 | 0.4335 | 0.3980 | 0.8010 | 0.8345 | 0.8375 | 0.6030 | 0.6215 | 0.3980 |
| 0.5% | 0.4150 | 0.4965 | 0.4865 | 0.8905 | 0.9100 | 0.9105 | 0.7235 | 0.7410 | 0.5085 |
| 0.6% | 0.5030 | 0.5925 | 0.6040 | 0.9335 | 0.9415 | 0.9435 | 0.8115 | 0.8205 | 0.6090 |
| 0.7% | 0.5660 | 0.6370 | 0.6855 | 0.9595 | 0.9645 | 0.9660 | 0.8655 | 0.8815 | 0.6960 |
| 0.8% | 0.6305 | 0.6900 | 0.7485 | 0.9810 | 0.9880 | 0.9890 | 0.9140 | 0.9210 | 0.7500 |
| 0.9% | 0.6915 | 0.7445 | 0.8340 | 0.9865 | 0.9860 | 0.9865 | 0.9445 | 0.9500 | 0.8680 |
| 1% | 0.6960 | 0.7780 | 0.8765 | 0.9920 | 0.9920 | 0.9925 | 0.9640 | 0.9665 | 0.8680 |
|  |  |  |  |  |  |  |  |  |  |
| False positive rate for MAF=.05 (N=12000) | | | | | | | | | |
| 0% | 0.052 | 0.0505 | 0.049 | 0.127 | 0.1905 | 0.1905 | 0.0395 | 0.05 | .058 |
|  |  |  |  |  |  |  |  |  |  |
| Note: Power to detect a GV that explains varying amounts of variance in the 2^nd^ of 2 latent factors.  Abbreviations are: *sum*: analysis of the sum across all phenotypes; *factor*: analysis of the factors score across all phenotypes calculated as Thompson scores; *MANOVA*: multivariate-analysis of variance with all phenpotypes as dependent variables; *Fisher*: Fisher combination test; *Fisher-L*: Lancaster’s weighted Fisher test; *Z*: Z-transform test; *Simes*: original Simes test; *TATES*: trait-based association test using extended Simes procedure.  Nphenotype =20, Nsubject=2000, Nsimulation=2000. | | | | | | | | | |
