# Supplementary material for: TATES: Efficient Multivariate Genotype-Phenotype Analysis for Genome-Wide Association Studies
Source: PLoS Genet. 2013 Jan 24;9(1):e1003235. doi: 10.1371/journal.pgen.1003235 (PMC3554627; doi:10.1371/journal.pgen.1003235)
Supplement: Table S11 — Power to detect GV (MAF = .5) in a 4-factor model, with 5 phenotypes per factor, factor loadings of .9, factorial correlations of .1, and GV effect specific to the 4th factor (Figure 1g. B1). (DOC) [file pgen.1003235.s012.doc]

| Table S11  Power to detect GV (MAF=.5) in a 4-factor model, with 5 phenotypes per factor, factor loadings of .9, factorial correlations of .1, and GV effect specific to the 4^th^ factor (Fig. 1g. B1) | | | | | | | | | |
| --- | --- | --- | --- | --- | --- | --- | --- | --- | --- |
|  | sum | factor | MANOVA | Fisher | Fisher-L | Z | Simes | TATES | MultiPhen |
| 0% | 0.0550 | 0.0470 | 0.0475 | 0.1190 | 0.1830 | 0.1845 | 0.0370 | 0.0575 | 0.0445 |
| 0.1% | 0.0910 | 0.1110 | 0.0935 | 0.2820 | 0.3385 | 0.3405 | 0.1300 | 0.1685 | 0.0995 |
| 0.2% | 0.1480 | 0.1785 | 0.1650 | 0.4385 | 0.4720 | 0.4735 | 0.2785 | 0.3300 | 0.1695 |
| 0.3% | 0.1875 | 0.2375 | 0.2070 | 0.5645 | 0.5580 | 0.5615 | 0.4060 | 0.4630 | 0.2180 |
| 0.4% | 0.2385 | 0.2885 | 0.3250 | 0.7035 | 0.6755 | 0.6820 | 0.5710 | 0.6255 | 0.3150 |
| 0.5% | 0.2725 | 0.3430 | 0.3680 | 0.7860 | 0.7210 | 0.7315 | 0.6695 | 0.7190 | 0.4005 |
| 0.6% | 0.3165 | 0.3710 | 0.4695 | 0.8590 | 0.7735 | 0.7845 | 0.7765 | 0.8105 | 0.4770 |
| 0.7% | 0.3750 | 0.3735 | 0.5820 | 0.9130 | 0.8460 | 0.8530 | 0.8585 | 0.8835 | 0.5520 |
| 0.8% | 0.4165 | 0.4225 | 0.6300 | 0.9395 | 0.8675 | 0.8770 | 0.9045 | 0.9225 | 0.6685 |
| 0.9% | 0.4585 | 0.4605 | 0.6900 | 0.9620 | 0.8965 | 0.9055 | 0.9365 | 0.9510 | 0.6955 |
| 1% | 0.4900 | 0.4740 | 0.7665 | 0.9785 | 0.9100 | 0.9230 | 0.9670 | 0.9755 | 0.7555 |
|  |  |  |  |  |  |  |  |  |  |
| False positive rate for MAF=.05 (N=12000) | | | | | | | | | |
| 0% | 0.0455 | 0.0585 | 0.0515 | 0.1245 | 0.1935 | 0.193 | 0.0395 | 0.059 | .045 |
|  |  |  |  |  |  |  |  |  |  |
| Note: Power to detect a GV that explains varying amounts of variance in the 4^th^ of 4 latent factors.  Abbreviations are: *sum*: analysis of the sum across all phenotypes; *factor*: analysis of the factors score across all phenotypes calculated as Thompson scores; *MANOVA*: multivariate-analysis of variance with all phenpotypes as dependent variables; *Fisher*: Fisher combination test; *Fisher-L*: Lancaster’s weighted Fisher test; *Z*: Z-transform test; *Simes*: original Simes test; *TATES*: trait-based association test using extended Simes procedure.  Nphenotype =20, Nsubject=2000, Nsimulation=2000. | | | | | | | | | |
