# Supplementary material for: TATES: Efficient Multivariate Genotype-Phenotype Analysis for Genome-Wide Association Studies
Source: PLoS Genet. 2013 Jan 24;9(1):e1003235. doi: 10.1371/journal.pgen.1003235 (PMC3554627; doi:10.1371/journal.pgen.1003235)
Supplement: Table S12 — Power to detect GV in a 4-factor model, with 5 phenotypes per factor, factor loadings of .9, factorial correlations of .1, and GV effect specific to one phenotype (Figure 1g. D1). (DOC) [file pgen.1003235.s013.doc]

| Table S12  Power to detect GV (MAF=.5) in a 4-factor model, with 5 phenotypes per factor, factor loadings of .9, factorial correlations of .1, and GV effect specific to one phenotype (Fig. 1g. D1) | | | | | | | | | |
| --- | --- | --- | --- | --- | --- | --- | --- | --- | --- |
|  | sum | factor | MANOVA | Fisher | Fisher-L | Z | Simes | TATES | MultiPhen |
| 0% | 0.0480 | 0.0510 | 0.0520 | 0.1085 | 0.1750 | 0.1715 | 0.0380 | 0.0560 | 0.0575 |
| 0.1% | 0.0600 | 0.0525 | 0.3390 | 0.1400 | 0.1980 | 0.1985 | 0.0765 | 0.1190 | 0.3475 |
| 0.2% | 0.0570 | 0.0525 | 0.7020 | 0.1570 | 0.2300 | 0.2310 | 0.1825 | 0.2345 | 0.7170 |
| 0.3% | 0.0565 | 0.0630 | 0.9080 | 0.2030 | 0.2460 | 0.2485 | 0.3035 | 0.3810 | 0.9160 |
| 0.4% | 0.0545 | 0.0735 | 0.9780 | 0.2310 | 0.2615 | 0.2640 | 0.4480 | 0.5355 | 0.9775 |
| 0.5% | 0.0590 | 0.0710 | 0.9975 | 0.2635 | 0.2870 | 0.2895 | 0.5770 | 0.6575 | 0.9965 |
| 0.6% | 0.0620 | 0.0690 | 0.9995 | 0.3045 | 0.2890 | 0.2925 | 0.6890 | 0.7560 | 0.9995 |
| 0.7% | 0.0690 | 0.0895 | 1.0000 | 0.3360 | 0.2860 | 0.2885 | 0.8030 | 0.8530 | 1.0000 |
| 0.8% | 0.0700 | 0.0920 | 1.0000 | 0.3595 | 0.3060 | 0.3100 | 0.8635 | 0.9060 | 1.0000 |
| 0.9% | 0.0730 | 0.1125 | 1.0000 | 0.4240 | 0.3165 | 0.3210 | 0.9115 | 0.9405 | 1.0000 |
| 1% | 0.0805 | 0.1105 | 1.0000 | 0.4840 | 0.3355 | 0.3460 | 0.9435 | 0.9605 | 1.0000 |
|  |  |  |  |  |  |  |  |  |  |
| False positive rate for MAF=.05 (N=12000) | | | | | | | | | |
| 0% | 0.0485 | 0.0545 | 0.044 | 0.109 | 0.1815 | 0.181 | 0.036 | 0.054 | .0445 |
|  |  |  |  |  |  |  |  |  |  |
| Note: Power to detect a GV that explains varying amounts of variance in one specific phenotype in the context of a 4-factor model.  Abbreviations are: *sum*: analysis of the sum across all phenotypes; *factor*: analysis of the factors score across all phenotypes calculated as Thompson scores; *MANOVA*: multivariate-analysis of variance with all phenpotypes as dependent variables; *Fisher*: Fisher combination test; *Fisher-L*: Lancaster’s weighted Fisher test; *Z*: Z-transform test; *Simes*: original Simes test; *TATES*: trait-based association test using extended Simes procedure.  Nphenotype =20, Nsubject=2000, Nsimulation=2000. | | | | | | | | | |
