# Supplementary material for: TATES: Efficient Multivariate Genotype-Phenotype Analysis for Genome-Wide Association Studies
Source: PLoS Genet. 2013 Jan 24;9(1):e1003235. doi: 10.1371/journal.pgen.1003235 (PMC3554627; doi:10.1371/journal.pgen.1003235)
Supplement: Table S15 — Power to detect GV in 1-factor Rasch model with factor loadings of .9747 (phenotypic intercorrelations .95), and GV effect on the factor. (DOC) [file pgen.1003235.s016.doc]

| Table S15  Power to detect GV (MAF=.5) in 1-factor Rasch model with factor loadings of .9747 (phenotypic intercorrelations .95), and GV effect on the factor | | |
| --- | --- | --- |
|  | Simes | TATES |
| 0% | 0.021 | 0.028 |
| 0.1% | 0.2035 | 0.242 |
| 0.2% | 0.406 | 0.4475 |
| 0.3% | 0.6105 | 0.6425 |
| 0.4% | 0.722 | 0.748 |
| 0.5% | 0.817 | 0.8445 |
| 0.6% | 0.8915 | 0.909 |
| 0.7% | 0.935 | 0.9445 |
| 0.8% | 0.9685 | 0.9735 |
| 0.9% | 0.9775 | 0.981 |
| 1% | 0.9855 | 0.9865 |
|  |  |  |
| Note: Power to detect a GV that explains varying amounts of variance in 1 latent factor.  Abbreviations are: *sum*: analysis of the sum across all phenotypes; *factor*: analysis of the factors score across all phenotypes calculated as Thompson scores; *MANOVA*: multivariate-analysis of variance with all phenpotypes as dependent variables; *Fisher*: Fisher combination test; *Fisher-L*: Lancaster’s weighted Fisher test; *Z*: Z-transform test; *Simes*: original Simes test; *TATES*: trait-based association test using extended Simes procedure.  Nphenotype =20, Nsubject=2000, Nsimulation=2000. | | |
