# Supplementary material for: TATES: Efficient Multivariate Genotype-Phenotype Analysis for Genome-Wide Association Studies
Source: PLoS Genet. 2013 Jan 24;9(1):e1003235. doi: 10.1371/journal.pgen.1003235 (PMC3554627; doi:10.1371/journal.pgen.1003235)
Supplement: Table S23 — Power to detect GV in 1-factor Rasch model with factor loadings of .75 (phenotypic intercorrelations .56), 10% blockwise missingness, and GV effect specific to one phenotype showing blockwise missingness. (DOC) [file pgen.1003235.s024.doc]

| Table S23  Power to detect GV (MAF=.5) in 1-factor Rasch model with factor loadings of .75 (phenotypic intercorrelations .56), 10% blockwise missingness, and GV specific to a phenotype showing this missingness | | | | | | | | | |
| --- | --- | --- | --- | --- | --- | --- | --- | --- | --- |
|  | Sum valid | Sum imputed | MANOVA  imputed | Simes  valid  unweighted | Simes valid  weighted | Simes imputed | TATES  valid  unweighted | TATES valid  weighted | TATES  imputed |
| 0% | 0.0565 | 0.056 | 0.052 | 0.039 | 0.032 | 0.0405 | 0.049 | 0.0445 | 0.0485 |
| 0.1% | 0.0585 | 0.0555 | 0.1395 | 0.0745 | 0.062 | 0.074 | 0.086 | 0.0755 | 0.0865 |
| 0.2% | 0.0495 | 0.05 | 0.2405 | 0.1275 | 0.1095 | 0.1285 | 0.1465 | 0.129 | 0.142 |
| 0.3% | 0.046 | 0.0515 | 0.3915 | 0.225 | 0.2015 | 0.2275 | 0.25 | 0.228 | 0.25 |
| 0.4% | 0.05 | 0.0555 | 0.56 | 0.339 | 0.3015 | 0.3355 | 0.3695 | 0.34 | 0.3655 |
| 0.5% | 0.056 | 0.0605 | 0.6935 | 0.429 | 0.4015 | 0.434 | 0.462 | 0.4305 | 0.459 |
| 0.6% | 0.0585 | 0.055 | 0.784 | 0.539 | 0.508 | 0.5355 | 0.5675 | 0.54 | 0.5645 |
| 0.7% | 0.054 | 0.0515 | 0.869 | 0.6705 | 0.639 | 0.668 | 0.6985 | 0.671 | 0.6965 |
| 0.8% | 0.0505 | 0.058 | 0.9215 | 0.736 | 0.7075 | 0.7345 | 0.758 | 0.738 | 0.755 |
| 0.9% | 0.043 | 0.044 | 0.947 | 0.8105 | 0.786 | 0.8105 | 0.8385 | 0.8125 | 0.836 |
| 1% | 0.0665 | 0.068 | 0.971 | 0.8645 | 0.8445 | 0.8635 | 0.879 | 0.8645 | 0.8755 |
|  |  |  |  |  |  |  |  |  |  |
| Note: Power to detect a GV that explains varying amounts of variance in 1 latent factor.  Abbreviations are: *sum valid*: analysis of the sum across all valid phenotypes, divided by the number of valid phenotypes; *sum imputed*: analysis of the sum across all phenotypes and missing values imputed by the mean of the phenotype; *MANOVA*: multivariate-analysis of variance with all phenotypes as dependent variables and missing values imputed by the mean of the phenotype; *Simes valid unweighted*: original Simes test based on all available data: all univariate tests, and thus the to-be- combined p-values, are all based on different sample sizes. *Simes valid weighted*: original Simes test based on all available data with p-values corrected for being based on different sample sizes by multiplying them by dfmax/dfj, where dfmax denotes the maximal number of degrees of freedom (i.e., sample size) of the 20 simulated phenotypes, and dfj denotes the number of degrees of freedom for the jth phenotype in the set of 1…20. *Simes imputed*: original Simes test based on mean imputed data. *TATES valid unweighted*: trait-based association test using extended Simes procedure based on all available data: all univariate tests, and thus the to-be- combined p-values, are all based on different sample sizes. *TATES valid weighted*: trait-based association test using extended Simes procedure based on all available data with p-values corrected for being based on different sample sizes by multiplying them by dfmax/dfj, where dfmax denotes the maximal number of degrees of freedom (i.e., sample size) of the 20 simulated phenotypes, and dfj denotes the number of degrees of freedom for the jth phenotype in the set of 1…20. *TATES imputed*: trait-based association test using extended Simes procedure based on mean imputed data.  The effect of missingness on power can be evaluated by comparing this Table to Table S4.  Nphenotype=20, Nsubject=2000, Nsimulation=2000. | | | | | | | | | |
