# Supplementary material for: TATES: Efficient Multivariate Genotype-Phenotype Analysis for Genome-Wide Association Studies
Source: PLoS Genet. 2013 Jan 24;9(1):e1003235. doi: 10.1371/journal.pgen.1003235 (PMC3554627; doi:10.1371/journal.pgen.1003235)
Supplement: Text S1 — Additional information on alternative methods to combine p-value information (Fisher combination test, Lancaster's weighted Fisher test, Z-transform test, original Simes test) and on calculation of confidence intervals for the p-values from the simulations. (DOC) [file pgen.1003235.s028.doc]

**Description additional methods used to combine p-value information**

*Fisher combination test*

The Fisher combination test (Fisher, 1954) is calculated as


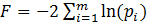
,

where *m* is the number of tests (i.e., phenotypes), and *p*_i_ is the p-value associated with the i^th^ phenotype. If the *m* tests are independent, this test statistic *F* is χ^2^-distributed with df=2*m*.

*Lancaster’s weighted Fisher test*

Lancaster’s weighted version of the Fisher combination test (Lancaster, 1961), is calculated as


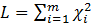
,

where *m* is the number of tests (i.e., phenotypes), *χ*^2^_i_ is the *χ*^2^ value corresponding to p_i_ when referring to a χ^2^-distribution with *df*=*N*_i_, and *N*_i_ is the sample size on which the test producing *p*_i_ was based. If the *m* tests are independent, the test statistic *L* is χ^2^-distributed with *df*=
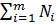
.

*Z-transform test*

The Z-transform test, also known as Stouffer’s method (Stouffer, 1949), is calculated as


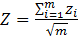
,

where *m* is the number of tests (i.e., phenotypes), and *Z*_i_ is the z-score corresponding to p_i_ when referring to a standard normal distribution. If the *m* tests are independent, the resulting test statistic *Z* is standard normally distributed[[1]](#footnote-2).

*Original Simes*

TATES, inspired by the paper by Li et al (2011), is an extension of this original Simes test (Simes, 1986). Let *p*_i_…*p*_m_ be the ascending p-values associated with the *m* phenotypes, and let *j* refer to the top *j* p-values where *j* runs from 1 to *m*. The original Simes test is calculated as


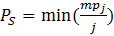
,

where *p*_j_ is the *j*^th^ p-value from the top. P_S_ corresponds to the smallest weighted p-value.

**Confidence intervals p-values simulations**

Note that the standard error of the ML estimator of the p-value is calculated as
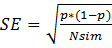
, where p denotes the percentage of significant tests observed in the simulations (nominal p-value) given the chosen α, and Nsim the total number of simulations. The 95% confidence interval for an unbiased nominal p-value when the GV-effect is actually zero (given α=.05 we expect p to be .05) thus corresponds to CI_95_=( p -1.96*SE, p +1.96*SE). Given Nsim=2000, CI_95_ thus equals .04-.06. That is, given α=.05, any observed overall nominal p-value outside the .04-.06 range should be considered incorrect: p-values < .04 suggest that the procedure is too conservative, while p-values >.06 suggest that the procedure is too liberal.

**References**

Fisher, R.A. (1954). Statistical methods for research workers. Twelfth Edition (New York: Hafner).

Lancaster, H. (1961). The combination of probabilities: an application of orthonormal functions. Australian Journal of Statistics, 3, 20–33.

Li, M-X., Gui, H-S., Kwan, J.S.H., & Sham, P.C. (2011). GATES: a rapid and powerful gene-based association test using extended Simes procedure. The American Journal of Human Genetics, 88 (3), 283-293.

Liptak, T. (1958). On the combination of independent tests. Magyar Tud. Akad. Mat. Kutato Int. Kozl. 3: 171-197.

Mosteller, F., & Bush, R.R. (1954). Selected quantitative techniques. In: Handbook of Social Psychology, Vol. 1 (G. Lindzey, ed.), 289-334. Addison-Wesley, Cambridge, Mass.

Simes, R. J. (1986). An improved Bonferroni procedure for multiple tests of significance. Biometrika 73, 751-754.

Stouffer, S.A., Suchman, E.A., DeVinney, L.C., Star, S.A. & Williams, R.M. Jr. 1949. The American Soldier, Vol. 1: Adjustment during Army Life. Princeton University Press, Princeton.

Whitlock, M.C. (2005). Combining probability from independent tests: the weighted Z-method is superior to Fisher’s approach. Journal of Evolutionary Biology, 18, 1368-1373.

1. Note that there is also a weighted version of the Z-transform test (Mosteller & Bush, 1954, Liptak, 1958, see also Whitlock, 2005). The results of this weighted test only differ from the results of the original Z-transform test if the to-be-combined tests are based on different sample sizes. Here, we wish to combine tests concerning multiple phenotypes measured within the same sample. Except when missingness patterns differ greatly across phenotypes (e.g., 5% missingness on phenotype 1, 50% on phenotype 2), it is unlikely that the sample sizes for each phenotype-based test differ such that the weighted Z-transform test yields results that differ greatly from those obtained with the unweighted test. Moreover, apart from the weighting, the weighted Z-transform test is based on the same calculation as the Z-transform test, which means that the tests have the same strengths and weaknesses. We therefore chose to discard the weighted Z-transform test in our simulations. [↑](#footnote-ref-2)
